# Humoral and cellular responses to repeated COVID-19 exposure in multiple sclerosis patients receiving B-cell depleting therapies: a single-center, one-year, prospective study

**DOI:** 10.3389/fimmu.2023.1194671

**Published:** 2023-06-28

**Authors:** Roberto Alfonso-Dunn, Jerry Lin, Joyce Lei, Jiayuan Liu, Morgan Roche, Antonia De Oliveira, Amol Raisingani, Anjali Kumar, Vanessa Kirschner, Grant Feuer, Michaela Malin, Saud A. Sadiq

**Affiliations:** Tisch Multiple Sclerosis Research Center of New York, New York, NY, United States

**Keywords:** multiple sclerosis, anti-CD20 therapy, B-cell depletion, COVID-19 vaccination, SARS-CoV-2 infection, omicron, anti-RBD antibody titer, T-cell response

## Abstract

Multiple sclerosis patients treated with anti-CD20 therapy (aCD20-MS) are considered especially vulnerable to complications from SARS-CoV-2 infection due to severe B-cell depletion with limited viral antigen-specific immunoglobulin production. Therefore, multiple vaccine doses as part of the primary vaccination series and booster updates have been recommended for this group of immunocompromised individuals. Even though much less studied than antibody-mediated humoral responses, T-cell responses play an important role against CoV-2 infection and are induced efficiently in vaccinated aCD20-MS patients. For individuals with such decoupled adaptive immunity, an understanding of the contribution of T-cell mediated immunity is essential to better assess protection against CoV-2 infection. Here, we present results from a prospective, single-center study for the assessment of humoral and cellular immune responses induced in aCD20-MS patients (203 donors/350 samples) compared to a healthy control group (43/146) after initial exposure to CoV-2 spike antigen and subsequent re-challenges. Low rates of seroconversion and RBD-hACE2 blocking activity were observed in aCD20-MS patients, even after multiple exposures (responders after 1st exposure = 17.5%; 2nd exposure = 29.3%). Regarding cellular immunity, an increase in the number of spike-specific monofunctional IFNγ^+^-, IL-2^+^-, and polyfunctional IFNγ^+^/IL-2^+^-secreting T-cells after 2nd exposure was found most noticeably in healthy controls. Nevertheless, a persistently higher T-cell response was detected in aCD20-MS patients compared to control individuals before and after re-exposure (mean fold increase in spike-specific IFNγ^+^-, IL-2^+^-, and IFNγ^+^/IL-2^+^-T cells before re-exposure = 3.9X, 3.6X, 3.5X/P< 0.001; after = 3.2X, 1.4X, 2.2X/P = 0.002, P = 0.05, P = 0.004). Moreover, cellular responses against sublineage BA.2 of the currently circulating omicron variant were maintained, to a similar degree, in both groups (15-30% T-cell response drop compared to ancestral). Overall, these results highlight the potential for a severely impaired humoral response in aCD20-MS patients even after multiple exposures, while still generating a strong T-cell response. Evaluating both humoral and cellular responses in vaccinated or infected MS patients on B-cell depletion therapy is essential to better assess individual correlations of immune protection and has implications for the design of future vaccines and healthcare strategies.

## 1 Introduction

The appearance and spread of SARS-CoV-2 (CoV-2), beginning in early 2020, have had a huge impact on society worldwide, with significant morbidity and mortality rates. Vaccines developed just one year into the pandemic are known to induce strong humoral and cellular responses and have provided an essential prevention tool for mitigating the impact of COVID-19 (1–5). Moreover, vaccination boosters (including monovalent or current bivalent doses) are necessary to provide enhanced humoral response potency and breadth with a resulting increase in immune protection (6–8). Still, concerns about loss of vaccine efficiency due to waning immunity and the appearance of immune-subversive CoV-2 variants (including the currently circulating and highly mutated sublineages of omicron VOC) have been sustained throughout the pandemic, especially for the most vulnerable population groups such as the elderly and immunocompromised individuals (9). Monitoring the level of immune protection provided by current vaccines in these groups is vital for adequate risk assessment, evaluation of healthcare strategies, and future vaccine development.

Immunocompromised individuals, including patients with different pathologies receiving immunosuppressive therapies, are considered more susceptible to severe disease and death from CoV-2 infection (9–12). Moreover, efficiency of COVID-19 vaccines is lower in immunocompromised individuals compared to the general healthy population and the use of vaccination boosters has been recommended in order to obtain higher immune protection (13–16). Of note, around 2.7% of the US adult population is considered immunosuppressed (17), encompassing a highly heterogenous group of conditions and pathologies as well as patients treated with a continuously expanding number of immunosuppressive therapies. At the immunological level, humoral and cellular immune responses to COVID-19 vaccinations, CoV-2 natural infections, and combinations of both are known to differ from one immunocompromised patient group to another. For instance, a normal response to vaccination has been described for untreated patients with multiple sclerosis (MS), although immune response shortcomings have been detected with specific disease-modifying therapy (DMT) treatments. This occurs most notoriously in patients on B-cell depletion therapy (BCDT) with limited humoral response, and individuals treated with fingolimod with reduced antibody and T-cell responses (18–20). Considering the high heterogenicity of conditions, immunosuppressive treatments, and antigen exposure experiences, an individualized assessment of the immune protection provided by vaccination or natural infection should be a priority for immunocompromised individuals.

Anti-CD20 monoclonal antibodies (including ocrelizumab, rituximab, and ofatumumab) are extensively used for the treatment of several cancers and autoimmune diseases, including lymphomas, systemic lupus erythematosus, rheumatoid arthritis, and MS (21). These antibodies bind and deplete CD20-expressing immature and mature/naïve B-cells and can therefore disrupt the *de novo* antibody production process in response to ongoing antigen exposure (22, 23), including in the context of CoV-2 natural infection and COVID-19 vaccination (24–27). Rates of CoV-2 infection in vaccinated immunocompetent individuals are known to be inversely correlated to levels of neutralizing antibodies (28, 29), thus explaining the observation that a higher CoV-2 breakthrough infection rate and longer viral incubation periods have been noticed in antiCD20-treated individuals (30–35). Regarding COVID-19 vaccination, a correlation between B-cell count and the production of anti-spike IgG levels has been observed in patients on BCDTs (36–38). In fact, several publications have estimated circulatory B-cell count number thresholds for achieving positive seroconversion (39–41). Considering the dynamics of anti-CD20-induced B-cell depletion and subsequent after-treatment repopulation, a time lapse longer than the standard 6-month schedule between therapy cycles (the currently approved regimen for MS treatment) is needed to favor seroconversion. Still, memory B cell repopulation kinetics vary considerably, and reaching normal values might not be possible even one year after discontinuing therapy (42, 43). Other factors that can play an important role in achieving seroconversion include total time of anti-CD20 treatment, age, sex, co-morbidities, and co-medications.

Antigen-specific T-cell responses have been described to play essential roles in the immune defense against viral infections, including SARS-CoV-2 (44). Moreover, current mRNA-based COVID-19 vaccines are known to induce strong poly-specific T-cell responses mediated by IFN+ or IL-2+ CD8^+^ and CD4^+^ Th1-cells (45), which have been shown to present higher durability (46–48) and cross-reactivity (49–51) against CoV-2 variants than serum neutralizing antibody responses. Of note, the role of T-cells in immune protection against CoV-2 infection and its correlation to vaccine protection has been much less studied compared to the contribution of antibodies. More research is needed, particularly in cases with limited humoral response.

Interestingly, several studies have shown the development of a robust T-cell response after infection or vaccination in aCD20-MS patients with severe B-cell depletion and failed seroconversion (52–57), suggesting a partial induction of the adaptive immune response. An essential question is how this T-cell-biased immunity performs in terms of real-world protection against virus-induced severe disease and death. Moreover, further analysis is urgently needed to determine the effect of antigen re-exposure on anti-CoV-2 humoral and cellular immune responses in aCD20-MS patients.

In this single-center, prospective, longitudinal study we characterize the adaptive immune responses of a cohort of multiple sclerosis patients treated with anti-CD20 (203 donors/350 samples collected) and healthy control individuals (43/146) after different COVID-19 exposure experiences (considering one, two, or three exposures and including vaccination, infection, or a combination of both). Antibody-based immune responses were studied by detection of IgG antibodies against the receptor binding domain (RBD) of CoV-2 spike protein and their RBD-hACE2 blocking activity, and cellular responses were assessed by measuring spike-specific IFNγ^+^-, IL-2^+^-, and polyfunctional IFNγ^+^/IL-2^+^-secreting T-cells using a FluoroSpot assay. Analysis included in this study provides information related to immune memory after antigen exposure, the effect of vaccination boosters and/or natural infections on humoral and cellular immunity, and the differential T-cell response against ancestral CoV-2 and omicron VOC. After first exposure (either through COVID-19 vaccination primary series or natural infection) we found only 17.5% of aCD20-MS patients with a positive humoral response (defined as positive for both anti-RBD IgG antibodies and RBD-hACE2 blocking activity) compared to 100% in healthy controls. Furthermore, limited humoral induction was still observed for aCD20-MS patients after re-exposure, with an increase in responders to only 29.3%. Recall immune response failure was also evident in aCD20-MS individuals who seroconverted after the first exposure, and in aCD20-naïve MS patients who received vaccination before initiation of treatment. Regarding cellular immunity, ancestral spike-specific T-cell responses increased after re-exposure, especially in healthy controls, but overall levels remained lower compared to the aCD20-MS cohort. Importantly, a similar 15-30% T-cell response decrease after peptide stimulation with an omicron (BA.2) version of spike protein was detected in both HC and aCD20-MS patients. In conclusion, the present study advances the characterization of the immune response to COVID-19 exposure in B-cell depleted multiple sclerosis patients treated with anti-CD20 and the results have implications for future vaccine design and application targeting this group of immunocompromised patients.

## 2 Materials and methods

### 2.1 Human subjects and study design

This prospective cohort study was initiated in 2021 and describes anti-SARS-CoV-2-spike-RBD immunoglobulin G (IgG) and CoV-2 spike-specific T-cell responses in the blood of multiple sclerosis patients receiving anti-CD20 monoclonal antibody-therapy (aCD20-MS; n = 203) compared to a healthy control group (HC; n = 43) after initial and subsequent (when available) exposure to CoV-2 spike protein (see next paragraph for definitions of the different exposures considered in this study). Also included is a small group of MS patients who had their first antigen exposure prior to starting anti-CD20 treatment (also known throughout the text as aCD20-naïve MS patients; n = 15). All participants were recruited from March 2021 to March 2022 and sample collection and analysis were performed by the same clinical and research personnel at Tisch MS Research Center of New York. For the HC cohort, healthcare workers donated blood samples routinely 4-5 times after initial CoV-2 exposure. For MS patients, samples were mostly provided (1 or 2 times) during the appointment for their scheduled anti-CD20 monoclonal antibody re-infusion with either rituximab or ocrelizumab. All aCD20-MS patients were actively treated with anti-CD20 monoclonal antibodies, with four patients switching from rituximab to ocrelizumab during this study. Naïve aCD20-MS patients included four untreated, three treated with natalizumab, three with dimethyl fumarate, one with interferon, and four receiving non-DMT treatments. For these MS patients, the first sample collection date coincided with their first anti-CD20 infusion. Information regarding CoV-2 infection and COVID-19 vaccination status as well as clinical data from patients were collected from questionnaires and medical records. Serologic and cellular data after a two-dose mRNA-based primary COVID-19 vaccination series have been partially published elsewhere (57).

Samples provided by participants were grouped based on exposure profiles considering vaccination, CoV-2 infection, and a combination of both (**Tables 1**, **S1**). Up to three spike protein exposure groups were considered in this study with the following profiles: i) “1st exposure” includes individuals (HC = 43; aCD20-MS = 154; aCD20-naïve MS = 15) receiving primary COVID-19 vaccination series or one CoV-2 episode; ii) “2nd exposure” comprises individuals (HC = 26; aCD20-MS = 109) receiving a vaccination booster or CoV-2 infection (breakthrough infection) after primary series, or a primary series after initial infection; iii) “3rd exposure” covers individuals (HC = 6; aCD20-MS = 11) with: a) a primary vaccination series and two boosters, b) CoV-2 infection with a subsequent primary series and one booster, c) primary series + breakthrough infection + booster, or d) primary series + booster + breakthrough infection. Longitudinal samples post exposure without re-challenge and 1st-to-2nd and 2nd-to-3rd exposure transitions were available for several individuals. Distribution of donors and samples per type of exposure, together with sample collection, anti-CD20 infusion, and exposure schedules are indicated in **Tables 1**, **S1** and throughout the text, figures, and figure legends.

**Table 1:** Clinical characteristics of study participants with first and second COVID-19 exposure.

	First EXPOSURE				Second EXPOSURE	
	HC	aCD20-MS	aCD20-naïve MS		HC	aCD20-MS
**Donors, n**	43	154	15	**Donors, n**	26	109
**Samples, n**	114	205	15	**Samples, n**	26	132
**Age, mean years (range)**	35.3 (23-65)	53.7 (28-82)	48.3 (24-75)	**Age, mean years [range]**	37.7 (23-59)	55.7 (27-77)
**Gender, n (%)**				**Gender, n (%)**		
Male	12 (27.9%)	52 (33.8%)	6 (40%)	Male	6 (23.1%)	34 (31.2%)
Female	31 (72.1%)	102 (66.2%)	9 (60%)	Female	20 (76.9%)	75 (68.8%)
**MS type, n (%)**				**MS type, n (%)**		
RRMS		82 (53.2%)	12 (80%)	RRMS		54 (49.5%)
SPMS		58 (37.7%)	2 (13.3%)	SPMS		42 (38.5%)
PPMS		14 (9.1%)	1 (6.7%)	PPMS		13 (12%)
**anti-CD20 therapy, n (%)**				**anti-CD20 therapy, n (%)**		
Ocrelizumab		104 (67.5%)		Ocrelizumab		77 (70.6%)
Rituximab		50 (32.5%)		Rituximab		32 (29.4%)
**CD19+ absolute count > 20 cell/μl, n (%)**		11 (7.1%)	14 (93.3%)	**CD19+ absolute count > 20 cell/μl, n (%)**		4 (3.7%)
**CD^+^ absolute count, mean cell/μl [reference range]**				**CD^+^ absolute count, mean cell/μl [reference range]**		
CD3^+^		1289 [708-2662]	1461 [708-2662]	CD3^+^		1322 [708-2662]
CD4^+^		936 [410-1604]	1012 [410-1604]	CD4^+^		922 [410-1604]
CD8^+^		349 [137-1003]	442 [137-1003]	CD8^+^		406 [137-1003]
**CD4^+^/CD8^+^ ratio [reference range]**		3.5 [0.9-4.2]	2.5 [0.9-4.2]	**CD4^+^/CD8^+^ ratio [reference range]**		3.6 [0.9-4.2]
**Vaccination, n (%)**				**Vaccinated + Booster, n (%)**		
BNT162b2/Comirnaty (Pfizer-BioNTech)	39 (90.7%)	82 (53.2%)	9 (60%)	**^#^Homologous**		
mRNA-1273/Spikevax (Moderna)		60 (39.0%)	4 (26.7%)	BNT162b2/Comirnaty (Pfizer-BioNTech)	5 (19.2%)	33 (30.3%)
Ad26.COV2.S (J&J-Janssen)		6 (3.9%)		mRNA-1273/Spikevax (Moderna)		31 (28.4%)
**CoV-2 infection, n (%)**	4 (9.3%)	6 (3.9%)	2 (13.3%)	Ad26.COV2.S (J&J-Janssen)		1 (0.9%)
**Time intervals, median days [IQR]**				**^#^Heterologous**		
^*^First exposure to collection	62 [57-65]	40 [27-70]	79 [48-155]	BNT162b2 + mRNA-1273	11 (42.3%)	9 (8.3%)
First exposure to last anti-CD20 infusion		133 [105-156]		mRNA-1273 + BNT162b2		1 (0.9%)
**Longitudinal samples (n donors/n samples)**				Ad26.COV2.S (J&J-Janssen) + mRNA-1273		2 (1.8%)
First exposure without re-exposure	31/102	49/100		**CoV-2 Infection and vaccination, n (%)**		
				Vaccinated + Infected	8 (30.8%)	7 (6.4%)
				Infected + Vaccinated	2 (7.7%)	25 (22.9%)
				**Time intervals, median days [IQR]**		
				^*^ Second exposure to collection	41 [30-62]	34 [24-52]
				First exposure to last anti-CD20 infusion		128 [93-154]
				Second exposure to last anti-CD20 infusion		155 [134-177]
				First to Second exposure	312 [277-329]	176 [95-195]
				**Longitudinal samples (n donors/n samples)**		
				Second exposure without re-exposure		23/46
				First to Second exposure	26/52	62/124

Clinical characteristics of healthy control individuals (HC) and MS patients on continuous anti-CD20 treatment (aCD20-MS) with first and second COVID-19 exposure. Also included is a group of MS patients not treated with anti-CD20 at the moment of first exposure (aCD20-naïve MS; only first exposure samples available).

*For donors with more than one longitudinal point, only the earliest point collected was considered.

^#^Homologous boosters are considered the same as the primary vaccine, and heterologous boosters are different from the primary vaccine.

IQR, interquartile range.

All vaccinations considered in this study were applied as intramuscular monovalent doses. First vaccination dose(s) applied to an individual (either during 1st exposure or 2nd exposure after infection) always constitutes a standard primary vaccination series [2-dose vaccine with a 3-to-4-week dosing regimen for mRNA-based vaccines BNT162b2 (Cominarty; Pfizer-BioNTech) and mRNA-1273 (Spikevax; Moderna) or a single-dose for adenovirus-based vaccine Ad26.COV2.S (J&J-Janssen)]. For this study, all primary series, regardless of the dosing regimen, are considered as a single exposure to the antigen. Subsequent vaccination boosters with mRNA- or adenovirus-based vaccines are also considered as individual exposures. The first primary vaccination series and first booster were applied in January and October 2021, respectively, for the HC control group and in December 2020 and May 2021 for the MS group. Information about the specific types of vaccines and boosters received by the 1st and 2nd exposure groups (including the homologous and heterologous vaccine booster combinations applied) are indicated in **Table 1**.

Only individuals with one COVID-19 infection episode were included in this study. Most self-reported infections took place during the 1-year period of sample collection, and a small number were convalescence COVID-19 cases from 2020. No sequencing information was collected regarding the specific CoV-2 variant involved in each infection.

### 2.2 Ethical approval

Ethical approval for this study was granted by the Western institutional review board (IRB)-approved protocol (STUDY NUM = 1305686; WIRB ID = 20211254). All study participants provided a written informed consent form before sample collection.

### 2.3 Serum and peripheral blood mononuclear cell isolation

Venous blood was collected from aCD20-MS patients and healthy control individuals at the Tisch MS Research Center of New York between March 2021 and March 2022 by the same clinical personnel. For aCD20-MS patients, most samples were provided during their scheduled anti-CD20 monoclonal antibody re-infusion date. Peripheral lymphocyte count analysis with absolute numbers of CD19^+^ B-cells, CD4^+^, and CD8^+^ T-cells was also performed before treatment infusion and sample collection. All sample processing was performed within 30 minutes after blood collection. For PBMC isolation, blood samples were collected into a Vacutainer^®^ CPT™ mononuclear cell preparation tube with sodium heparin anticoagulant and a FICOLL™ Hypaque™ solution (BD Biosciences, #362753), mixed by inversion 5-8 times, and centrifugated for 15 minutes at 1500 x g at room temperature (RT). Mononuclear cells were recovered below the plasma layer, washed once with PBS, counted, and resuspended with CryoStor^®^ CS10 preservation media (STEMCELL, #07930). Cells were stored in cryogenic tubes (Thermo Scientific™, #5000-1012) at a concentration of 1.5-3x10^6^ cells/mL and maintained in liquid nitrogen until use for T-cell-based assays. PBMC samples were only obtained from a subgroup of study participants. Serum samples were obtained from the same blood draw and processed using a Vacutainer SST™ tube (BD Biosciences, #367988). Aliquots of serum were immediately stored at -80 °C.

### 2.4 Detection of the antibody-mediated spike RBD–hACE2 blocking activity

The serum capacity to block the angiotensin-converting enzyme-2 (hACE2) interaction with the receptor binding domain (RBD) of CoV-2 spike protein was detected using the commercial SARS-CoV-2 Surrogate Virus Neutralization Test cPass kit (GenScript Biotech). Serum samples diluted 1/10 were first incubated with HRP-conjugated RBD recombinant protein, and circulating antibodies with functional neutralizing capacity were detected by application of the mix onto ELISA plate wells coated with purified hACE2 protein. All samples and controls were tested in duplicates and percent inhibition of RBD-hACE2 binding was calculated using the following equation: % inhibition = $\left(1-\left[\frac{\text{OD of serum}+\text{RBD}}{\text{OD of negative control}+\text{RBD}}\right]\right)$ x 100. Based on the manufacturer’s instructions, a percent inhibition of ≥ 30% was used as a cutoff to indicate the presence of SARS-CoV-2 RBD-interacting antibodies blocking RBD-hACE2 interaction.

### 2.5 Detection of immunoglobulin G antibodies against spike RBD using an ELISA assay

Serum IgG binding antibodies to RBD of spike protein were quantified using an enzyme-linked immunosorbent assay (ELISA) as previously described (57). Recombinant ancestral-based CoV-2 RBD protein (Raybiotech, #230-30162-100) was diluted to a final concentration of 0.5 µg/mL, coated onto 96-well, high-binding surface EIA/RIA Assay Microplates (Corning, #CLS3361), and incubated at 4°C overnight. After washing, plates were blocked with PBS containing 1% non-fat dry milk at RT for 1 hour. Serum samples were diluted 1:1600 with blocking solution and 100 μL per well were added considering technical duplicates. A serum sample from a participant with confirmed COVID-19 diagnosis was used as positive control and applied in each plate to normalize data points within and between plates. After a 1-hour incubation at RT, plates were washed five times with PBST and RBD-specific antibodies were detected using a HRP-conjugated Goat anti-Human IgG (H+L) cross-adsorbed secondary antibody (ThermoFisher, #A18811) diluted 1:20000 with blocking solution. After one hour at RT, plates were incubated with tetramethylbenzidine (TMB) Substrate Solution (ThermoFisher, #N301) for 10 min prior to stopping the reaction with 0.16 M sulfuric acid Stop Solution (ThermoFisher, #N600). Plates were read on a BioTek Synergy HT microplate reader at 450 nm within 30 min of stopping the reaction. Optical density (OD) is calculated as the absorbance at 450 nm and data are presented as relative to a known positive serum sample control. A positivity cut-off value (limit of quantification) was established by considering the mean anti-RBD + S.D. value obtained from pre-2020 samples (n = 30). Humoral responders were defined as individuals with positive seroconversion based on our ELISA assay (> 0.18) and positive RBD-hACE2 blocking activity (> 30%). All humoral assays were performed with the use of RBD of spike protein derived from the ancestral SARS-CoV-2 WA1/2020 strain.

### 2.6 Detection of spike-specific IFNγ^+^, IL-2^+^, and IFNγ^+^/IL-2^+^-secreting T-cells using a FluoroSpot assay

Simultaneous *ex vivo* detection of interferon gamma (IFNγ)^+^-, interleukin-2 (IL-2)^+^-, and IFNγ^+^/IL-2^+^-secreting T-cells from collected PBMC samples was done using a FluoroSpot assay. Frozen PBMCs were thawed and immediately treated with Anti-Aggregate Wash™ Solution (CTL, #AA). 2-3x10^5^ cells resuspended with warm serum-free CTL-Test™ Medium (ImmunoSpot) were plated per well in a M96 FluoroSpot plate (ImmunoSpot) and incubated with a peptide pool spanning the entire open reading frame of the ancestral strain (Wuhan-Hu-1) spike protein (15-mer with 11 amino acid overlap; JPT, PepMix™ #PM-WCPV-S; 1 μg/mL) for 24 hours at 37 °C. In addition, incubations with DMSO (0.4%; representing DMSO content in peptide pools), or Ultra SuperStim Pool (JPT, #PM-CEFX; 1 μg/mL) were included as negative and positive controls respectively. To determine T-cell response against omicron spike protein, PBMCs were stimulated in parallel with peptide pools covering the entire CoV-2 spike protein sequence of the ancestral strain or the omicron VOC (BA.2) (JPT, #PM-SARS2-SMUT09-1; 1 μg/mL). Data for the ancestral and omicron stimulations from the same donor were obtained in the same assay. Co-stimulation with anti-CD28 (ImmunoSpot; 100 ng/mL) was included for all incubations and duplicate or triplicate wells were applied per stimulation. Fluorescent spots resulting from cells secreting IFNγ^+^, IL-2^+^, or IFNγ^+^/IL-2^+^ were counted using a Cellular Technology Limited S6 Universal Analyzer and data processed with ImmunoSpot^®^ 7.0 software. Counting parameters were set optimally for each filter individually and then a pairing algorithm using the center of mass distance for each spot was used to determine co-expressors. For each sample, the mean spot forming units (SFU) obtained from DMSO incubations was subtracted from the peptide stimulations and final data was expressed as ΔSFU per million PBMCs. Samples with less than 20 IFNγ^+^ and IL-2^+^ SFU per 10^6^ cells in CEFX stimulations were not considered in this study. For ancestral spike-specific stimulations, a value of (mean + 2xS.D.) obtained from unexposed individuals (pre-2020 and more recent unvaccinated/non-infected individuals) was used as the lower limit to indicate a positive response in the test group (57).

### 2.7 Quantification and statistical analysis

All quantifications and statistical analyses were performed with GraphPad Prism v9 (GraphPad Software). Non-parametric spearman’s correlation coefficient was calculated to detect possible associations between humoral (relative anti-RBD IgG OD) and T-cell (ΔSFU per million PBMC) responses. For paired comparisons, statistical analysis was performed using Wilcoxon matched-pairs signed rank, whereas the Mann–Whitney U test was applied to compare two independent data sets. Multiple data were analyzed with Kruskal–Wallis test and followed by Dunn’s multiple comparisons test. Scatter plots showing geometric mean with geometric S.D. are shown in figures. Time intervals between exposures and sample collections, and exposures and last anti-CD20 infusions, are indicated throughout the publication as median with interquartile range (IQR). P-values< 0.05 were considered statistically significant. Details related to significance are also noted in text and figure legends.

## 3 Results

### 3.1 Study characteristics

A single-center prospective study was organized to determine the immune response induced by COVID-19 vaccination and natural CoV-2 infection in MS patients actively treated with monoclonal anti-CD20 antibodies (ocrelizumab or rituximab) (n = 203). A healthy group of clinical workers (n = 43) and MS patients who had their first antigen exposure prior to starting treatment (aCD20-naïve MS; n = 15) were included in the study as controls (**Table 1**). Unique and longitudinal samples from participants were organized based on the number of CoV-2 antigen exposures (up to three exposures) considering vaccinations, natural infections, and a combination of both (**Tables 1**, **S1**). Participants with a single “1st exposure” (HC = 43; aCD20-MS = 154; aCD20-naïve MS = 15) included individuals vaccinated with a primary series [either a 2-dose mRNA-based vaccine (mRNA-1273 or BNT162b2) or a single-dose adenovirus-based vaccine (Ad26.COV2.S)], or individuals who experienced a single COVID-19 episode. Participants who received a “2nd exposure” (HC = 26; aCD20-MS = 109) were re-exposed to CoV-2 spike antigen either through an additional single-dose vaccination (vaccination booster) or natural CoV-2 infection (breakthrough infection) after a primary series, or a vaccination primary series after initial infection. Finally, a “3rd exposure” (HC = 6; aCD20-MS = 11) was observed for a small number of participants and included the following profiles (accrual of exposures indicated in chronological order): primary series + 2 vaccination boosters (aCD20-MS = 1); CoV-2 infection + primary series + single-dose vaccination booster (HC = 1; aCD20-MS = 8); primary series + breakthrough infection + single-dose vaccination booster (HC = 2; aCD20-MS = 2); and primary series + vaccination booster + breakthrough infection (HC = 3). In addition, paired longitudinal samples covering 1st to 2nd exposure transition were available for 26 HC and 62 aCD20-MS donors, and 2nd to 3rd exposure for nine patients.

For most HC participants, samples were collected at approximately 60, 90, 150, and 240 days after 1st exposure, 40 after 2nd exposure, and 30 after 3rd exposure. The median time interval (expressed as days [IQR]) between 1st-to-2nd and 2nd-to-3rd exposures was 312 [277-329] and 71 [33-184] days, respectively (**Tables 1**, **S1**). For aCD20-MS patients, sample collections usually coincided with their scheduled anti-CD20 infusion treatments. In fact, consecutive infusion treatments and sample collection dates (when available) occurred close to 180 days apart in concordance with the recommended 6-month anti-CD20 therapy schedule for MS treatment. This highlights an overall adherence to a standard treatment regimen within our aCD20-MS cohort throughout the duration of this study. In general, patients were more likely to receive CoV-2 antigen exposures during the last months of their inter-infusion time lapse (median days [IQR] between exposure and last anti-CD20 infusion: 1st exposure cohort = 133 [105-156]; 2nd = 155 [134-177]; 3rd = 162 [129-168]), probably intended to favor B-cell repopulation, and thus increased probability of seroconversion after vaccination. The restriction due to anti-CD20 treatment scheduling in the aCD20-MS group was also reflected in the time interval between exposures (median days [IQR] between exposures: 2nd to 1st exposure in 2nd exposure cohort = 176 [95-195]; 3rd to 2nd in 3rd exposure cohort =196 [154-212]) and the time of collection/infusion after exposure (median days [IQR] between exposure and closest sample collection date: 1st exposure cohort = 40 [27-70]; 2nd = 34 [24-52]; 3rd = 27 [22-44]. Importantly, for the majority of aCD20-MS participants, normal T-cell count values within reference range but with highly reduced CD19^+^ B-cell levels (< 20 cells per µL) were detected in routine blood sample analysis performed before treatment infusion and sample collection (**Tables 1**, **S1**).

### 3.2 Lower rate of seroconversion and RBD-hACE2 binding blocking activity in aCD20-MS patients compared to healthy controls after first exposure to CoV-2 spike protein

**Figure 1:**
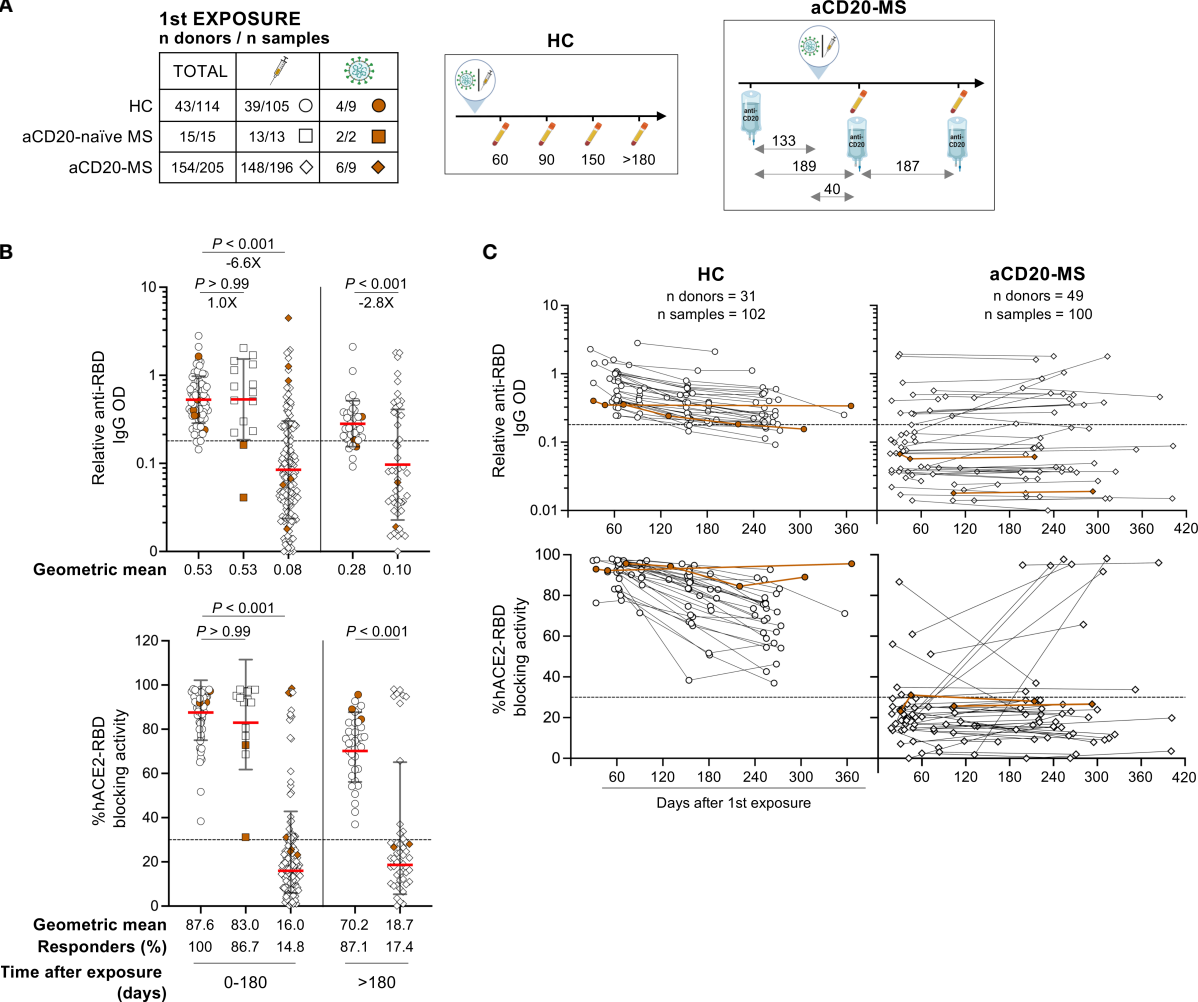
Deficient humoral response to first CoV-2 spike protein exposure in aCD20-MS patients compared to healthy controls. **(A–C)** Serum samples were collected from healthy individuals (HC, circles: n donors = 43/n samples = 114) and MS patients on continuous anti-CD20 treatment (aCD20-MS, diamonds: n donors = 154/n samples = 205) who were exposed once to CoV-2 spike protein [either through COVID-19 primary vaccination series (white) or CoV-2 infection (brown)]. A group of MS patients not treated with anti-CD20 at the moment of first exposure was also included (aCD20-naïve MS, squares: n donors = 15/n samples = 15). RBD IgG levels and antibody mediated RBD-hACE2 blocking activity were detected using an ELISA assay and the SARS-CoV-2 Surrogate Virus Neutralization Test cPass™ kit (GenScript), respectively. Dotted horizontal lines inside graphs indicate positivity cut-off levels for each assay. **(A)** Distribution of donors and samples grouped based on COVID-19 exposure profile (table, left) and illustrations (right) depicting the timescale of exposure and sample collection for HC and aCD20-MS groups. For aCD20-MS patients, the median days between sample collections, first COVID-19 exposure, and ongoing anti-CD20 infusion dates are indicated inside the diagram. All icons used in tables and illustrations were generated with BioRender. **(B)** Relative anti-RBD IgG OD values (top) and percentage hACE2/RBD blocking activity (bottom) for all first exposure samples grouped based on collection time range (0 to 180 and > 180 days). Percentage of responders (defined as individuals positive for both anti-RBD IgG and blocking activity) are indicated below the graphs. Data are presented as geometric mean (red line) with geometric S.D., and differences between groups were analyzed using the Kruskal-Wallis test (0 to 180 days samples) and the Mann-Whitney U test (> 180). Significant P values and fold changes are shown above each data set. **(C)** Longitudinal presentation of relative anti-RBD IgG OD values (top) and percentage hACE2/RBD blocking activity (bottom) in HC (102 samples from 31 donors) and aCD20-MS individuals (100 samples from 49 donors). Each figure dot represents a single sample with at least two antibody measurements per donor after first exposure. Longitudinal samples are linked with a solid line.

Previously, we observed a reduced rate of seroconversion in a group of anti-CD20 treated MS patients with severe B-cell depletion within 6 months after a 2-dose cycle mRNA-based COVID-19 vaccination (57). To gain a broader insight into the humoral immune memory response to an initial exposure to CoV-2, we expanded our study to include CoV-2 convalescent individuals and longitudinal time points (**Table 1** and **Figure 1A**). Serum Immunoglobulin G (IgG) levels were measured against the receptor binding domain (RBD) of the SARS-CoV-2 ancestral spike protein using an ELISA assay. In addition, the RBD-human cellular receptor angiotensin-converting enzyme 2 (hACE2) blocking activity of these antibodies was measured using a surrogate virus neutralization assay. RBD is essential for the initial interaction of the CoV-2 virion to the host cell and represents the main target of numerous neutralizing antibodies generated after CoV-2 infection and mRNA-based vaccination (58, 59).

Within 6 months after first exposure, lower levels of anti-RBD IgG (-6.6X; P< 0.001) and %hACE2-RBD blocking activity (P< 0.001) were detected in aCD20-MS patients compared to healthy controls (**Figure 1B**). Positive humoral response, defined as positive seroconversion response and hACE2-RBD blocking activity, was observed in 100% of healthy controls and 14.8% of MS patients receiving anti-CD20 therapy infusion on a median of 133 IQR [105-156] days before exposure (**Table 1** and **Figure 1A**). In addition, 86.7% of MS patients exposed to CoV-2 spike antigen before initiation of anti-CD20 treatment (aCD20-naïve MS) had a positive response with relative anti-RBD OD values (geometric mean: 0.53) similar to the HC cohort (0.53; P> 0.99). Humoral response from samples collected after 6 months and decay profiles considering longitudinal and cross-sectional samples highlight a decreased antibody-mediated immune response in HC individuals similar to that previously described (**Figures 1B, C**, **S1A**) (5). In contrast, only 17.5% of aCD20-MS patients who received continuous drug treatment generated humoral response at any given time after first exposure. Moreover, no significant correlations were found between last anti-CD20 infusion to exposure interval and antibody levels or RBD-hACE2 blocking activity (**Figure S1B**). Collectively, these results demonstrate a severe deficiency in the ability to develop a humoral response after a first exposure to CoV-2 spike antigen in MS patients actively treated with anti-CD20 therapy.

### 3.3 Limited humoral response in aCD20-MS after multiple exposures to CoV-2 spike protein

In the context of a waning humoral response 6 months after primary vaccination series or natural CoV-2 infection and the emergence of highly CoV-2 immune-evasive variants, the recommendation of a third immunization dose based on the ancestral Wuhan-Hu-1 spike sequence was suggested in the second half of 2021 (47, 60–63). Timely spaced re-exposure after first antigen encounter is characterized by an increase in antibody quantity and quality with enhanced antigen binding, neutralizing potency, and breadth. This improvement is known to be driven by the re-activation and expansion of memory B-cell clones as well as the emergence of new germlines.

**Figure 2:**
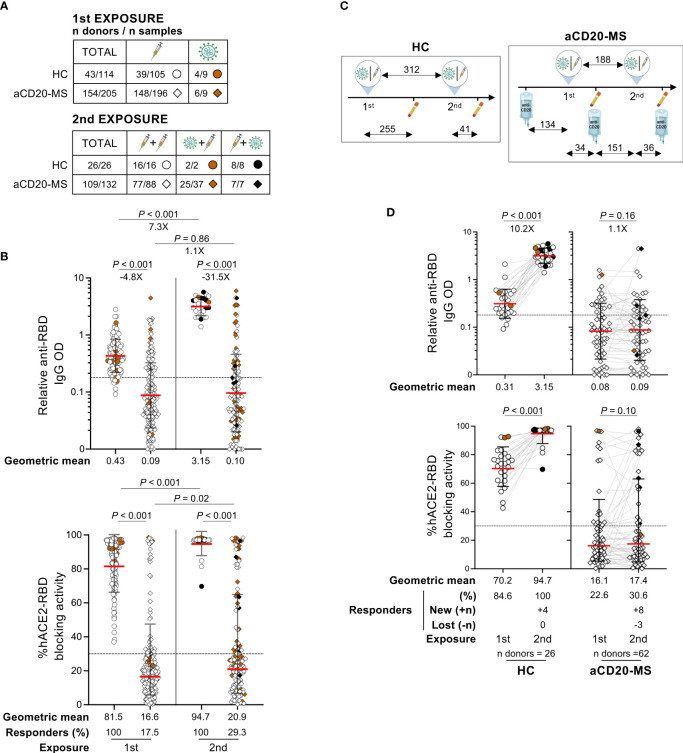
Deficient humoral response after second CoV-2 spike protein exposure in aCD20-MS patients. **(A–D)** Serum samples were collected from HC individuals (circles) and aCD20-MS patients (diamonds) exposed once (HC: n donors = 43/n samples = 114; aCD20-MS: 154/205) or twice (HC: n donors = 26/n samples = 26; aCD20-MS: 109/132) to CoV-2 spike protein. **(A, B)** includes all grouped samples while **(C, D)** presents only paired longitudinal samples. RBD IgG levels and antibody mediated RBD-hACE2 blocking activity were detected using an ELISA assay and the SARS-CoV-2 Surrogate Virus Neutralization Test cPass™ kit (GenScript), respectively. Dotted horizontal lines inside graphs indicate positivity cut-off values for each assay. **(A)** Tables showing distribution of total donors and samples grouped based on COVID-19 exposure profile. **(B)** Relative RBD IgG OD values and percentage hACE2-RBD blocking activity after first and second antigen exposures in HC individuals and aCD20-MS patients. Data from grouped samples is presented as geometric mean (red line) with geometric S.D., and differences between first and second exposures within and between cohorts were calculated using a Mann-Whitney U test. Percentage of individuals with positive humoral response are indicated below the graphs and significant P values and fold changes are shown above each data set. **(C)** Illustrations depicting the timescale of exposures and sample collections for each group only considering participants with paired longitudinal samples (numbers indicate median days for each time lapse). **(D)** Longitudinal presentation of humoral response after COVID-19 re-exposure in HC (n = 26) and aCD20-MS patients (n = 62). Relative RBD IgG OD values and % RBD-hACE2 blocking activity are shown before and after second exposure to CoV-2 spike protein. Data are presented as geometric mean (red line) with geometric S.D., and differences between first and second exposure within each cohort were calculated using a two-tailed Wilcoxon paired test. Percentage of individuals with positive humoral response (including number of new and lost responders after re-exposure) are indicated below the graphs and significant P values and fold changes are shown above each data set.

Having noticed a low antibody response rate in aCD20-MS patients after initial antigen exposure, we decided to focus on the effect of a subsequent re-exposure. Under consideration for this analysis were participants with diverse immunological experiences, including individuals i) receiving vaccination boosters, ii) infected after vaccination (breakthrough infection), and iii) vaccinated after COVID-19 convalescence (**Table 1** and **Figure 2A**). A strong increase in the magnitude of spike RBD-specific IgG OD in healthy controls was observed at a median of 41 days [IQR 30-62] after a second exposure (median days [IQR] between 1st and 2nd exposures: 312 [277- 329]) with a 7.3X (P< 0.001) geometric mean fold change compared to levels after first exposure (**Figure 2B** showing all longitudinal points after 1st exposure grouped). The quantitatively superior humoral response from a 2nd antigen exposure in HC individuals was also evident when compared to the highest anti-RBD IgG level detected within 60 days after first exposure (geometric mean fold change: 4X; P = 0.02) (**Figure S2A**). Similar trends were observed for the antibody-mediated RBD-hACE2 blocking activity. In contrast, aCD20-MS patients receiving a vaccination booster or undergoing infection at a median of 176 days [95-195] post-1st exposure and 155 days [134-177] since their last anti-CD20 infusion presented minimal improvement of their humoral response (2nd to 1st exposure anti-RBD IgG geometric fold change: 1.1X; P = 0.86) (**Figure 2B**), with the difference in antibody levels between cohorts much more apparent after re-exposure (aCD20-MS vs HC anti-RBD IgG geometric fold change: after 1st exposure = -4.8X; P< 0.001/after 2nd exposure = -31.5X; P< 0.001). Overall, the percentage of positive responders detected at any given time before and after second exposure was of 100% at both times for HC individuals and 17.5% (before) and 29.3% (after) for aCD20-MS patients. Similar limited antibody-mediated immune response was observed in a small group of patients exposed for a third time to CoV-2 antigen (**Table S1** and **Figure S3A**). Notably, a comparable trend was apparent when considering longitudinal samples before and after re-exposure (**Figures 2C, D**, **S3B**). In terms of immunological memory after a second antigen exposure, paired longitudinal data in aCD20-MS patients showed variable decay kinetics over time for positive responders (**Figure S2B**). Interestingly, a small fraction of these patients presented a delayed humoral response with higher antibody levels and hACE2-RBD blocking activity at later times of collection, similar to that observed after first exposure (**Figure 1C**) and in accordance with a previous study (53).

Finally, failed recall response after second exposure was observed for 11 out of 12 aCD20-MS patients receiving continuous treatment with a positive humoral response after first exposure (**Figure S4A**), and for three out of four participants with their first antigen contact occurring with normal levels of peripheral CD19^+^ B-cells prior to initiation of anti-CD20 therapy (aCD20-naïve MS) (**Figure S4B**). In both groups, similar or lower anti-RBD IgG OD values were found for most participants after a second exposure. Overall, aCD20-MS patients showed a persistently reduced humoral response even after multiple CoV-2 spike antigen exposures and defective recall response in previously seroconverted individuals.

### 3.4 Increased spike-specific T-cell response after antigen re-exposure and lack of correlation between humoral and cellular responses

Extensive work on the characterization of T-cell responses to SARS-CoV-2 infection and COVID-19 vaccination has established an important role for the cellular adaptive immune response in providing protection against SARS-CoV-2 infection (44). We previously found a higher number of spike-specific IFNγ^+^, IL-2^+^, and polyfunctional IFNγ^+^/IL-2^+^ T-cells, with enhanced proliferative capacity, in aCD20-MS patients compared to a control group after primary vaccination series with two doses of mRNA-based COVID-19 vaccines (57). Increased cellular response in vaccinated aCD20-MS patients after first exposure has been observed in some studies (53–55), but not in others (20, 64). Moreover, the effect of antigen re-exposure in further boosting this response has not been extensively studied. Proper monitoring and characterization of T-cell responses after antigen exposure(s) in aCD20-MS patients is particularly important due to their potential for developing a severely impaired humoral response.

**Figure 3:**
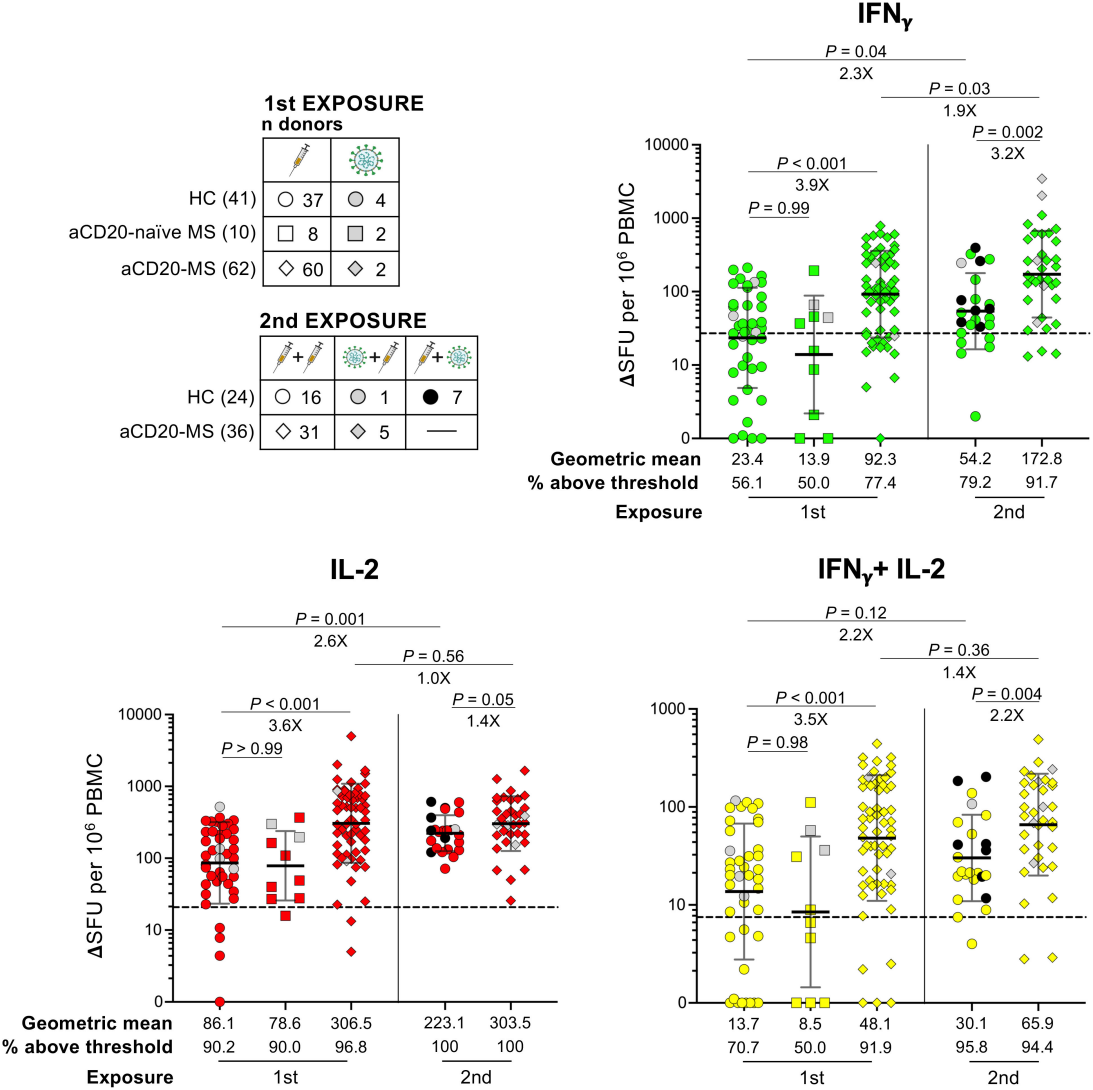
Ancestral spike-specific IFNγ^+^, IL-2^+^, and IFNγ^+^/IL-2^+^ T-cell responses after first and second COVID-19 exposure in aCD20-MS cohort compared to healthy controls. PBMCs isolated from HC and aCD20-MS patients after first and second antigen exposure were stimulated with a 15-mer peptide pool spanning the whole ancestral spike protein (1μg/mL) during 24 hours at 37 °C. A FluoroSpot assay was used to determine the number of spike-specific IFNγ^+^(green)-, IL-2^+^(red)-, and IFNγ^+^/IL-2^+^(yellow)-secreting T-cells (expressed as ΔSFU per 10^6^ PBMC). Cytokine^+^-secreting T-cells for HC (circles), MS patients not treated with anti-CD20 at the moment of first exposure (aCD20-naïve MS, squares) and aCD20-MS patients (diamonds) after first (HC: n donors = 41; aCD20-naïve MS: 10; aCD20-MS: 62) or 2nd COVID-19 exposure (HC: 24; aCD20-MS: 36). Distribution of total donors based on COVID-19 exposure profile is indicated in left top tables. Geometric means and the fraction of individuals above positivity threshold are presented below the graphs, and significant P values and fold changes are shown above each data set. Data are presented as geometric mean (black line) ± geometric S.D., and differences between cohorts after first exposure were analyzed using the Kruskal-Wallis test. Differences between cohorts after second exposure and between exposures within cohorts were analyzed using the Mann-Whitney U test. Dotted lines in graphs indicate mean + 2xS.D. positivity thresholds obtained from unexposed controls (IFNγ^+^ = 27, IL-2^+^ = 20.8, IFNγ^+^/IL-2^+^ = 7.5) (57). See also **Figure S5**.

To characterize the cellular immune response, we began examining the effect of a first antigen exposure in a subgroup of HC individuals and aCD20-MS patients (**Figure 3**, 1st exposure). To this end, PBMCs were stimulated *ex vivo* with a 15-mer peptide pool overlapping the whole ancestral spike protein and the detection of monofunctional IFNγ^+^-, IL-2^+^-, and polyfunctional IFNγ^+^/IL-2^+^-secreting T-cells was achieved using a FluoroSpot assay (see **Figure S5** for representative FluoroSpot data). As control, we performed simultaneous stimulations with CEFX, a positive control peptide pool with immunodominant peptides from common viruses, including Epstein-Barr, influenza, and cytomegalovirus. In addition to healthy control individuals (n = 41) and aCD20-MS patients (n = 62), a group of MS patients not treated with anti-CD20 therapy (aCD20-naïve MS) was also included (n = 10). As previously described (57), a higher geometric mean ΔSFU per 10^6^ PBMC values was detected in aCD20-MS patients (geometric mean: IFNγ^+^ = 92.3, IL-2^+^ = 306.5, IFNγ^+^/IL-2^+^ = 48.1) compared to healthy controls (23.4, P< 0.001; 86.1, P< 0.001; 13.7, P< 0.001). This difference was also reflected when considering the percentage of participants above a positivity threshold level obtained after stimulation of PBMCs isolated from pre-2020 and un-exposed individuals with the ancestral-spike peptide pool (HC: IFNγ^+^ = 56.1%, IL-2^+^ = 90.2%, IFNγ^+^/IL-2^+^ = 70.7%; aCD20-MS: 77.4%, 96.8%, 91.9%) (57). Furthermore, the number of cytokine-expressing T-cells in aCD20-naïve MS patients was similar to HC (geometric mean: IFNγ^+^ = 13.9, P = 0.99; IL-2^+^ = 78.6, P> 0.99; IFNγ^+^/IL-2^+^ = 8.5, P = 0.98), suggesting that the observed increase in cellular response after first exposure is specific to treatment with anti-CD20 therapy. Interestingly, and contrary to the observed spike-specific T-cell response, CEFX-specific response was lower in aCD20-MS patients compared to HC, reaching significant difference for IL-2^+^ (P = 0.006) and IFNγ^+^/IL-2^+^ (P = 0.004) (**Figure S6**, 1st exposure).

Next, we determined the effect of a second exposure on the spike-specific T-cell response (**Figure 3**, 2nd exposure). An increase in geometric mean spot counts was observed for the HC group after antigen re-exposure (geometric mean fold increase: IFNγ^+^ = 2.3X, P = 0.04; IL-2^+^ = 2.6X, P = 0.001; IFNγ^+^/IL-2^+^ = 2.2X, P = 0.12). In contrast, a more modest boost in the number of IFNγ^+^ T-cells was detected in the aCD20-MS group, with no significant changes in IL-2^+^- and IFNγ^+^/IL-2^+^- secreting cells (IFNγ^+^ = 1.9X, P = 0.03; IL-2^+^ = 1.0X, P = 0.56; IFNγ^+^/IL-2^+^ = 1.4X, P = 0.36). These variations were spike-specific since no significant differences were detected after CEFX stimulation (**Figure S6**). Even though a stronger increase in cellular response after a second instance of COVID-19 exposure was observed in healthy controls, the aCD20-MS cohort maintained higher mean spike-specific cytokine^+^-secreting T-cell numbers before (geometric mean aCD20-MS vs HC fold increase: IFNγ^+^ = 3.9X, P< 0.001; IL-2^+^ = 3.6X, P< 0.001; IFNγ^+^/IL-2^+^ = 3.5X, P< 0.001) and after re-exposure (3.2X, P = 0.002; 1.4X, P = 0.05; 2.2X, P = 0.004). Overall, the percentage of HC individuals above threshold signal increased from 56.1% (IFNγ^+^), 90.2% (IL-2^+^), and 70.7% (IFNγ^+^/IL-2^+^) before re-exposure to 79.2%, 100%, and 95.8% after. For aCD20-MS patients, a small increase was observed in the number of IFNγ^+^-secreting T-cells (from 77.4% before to 91.7% after), with almost full positivity before and after re-exposure for IL-2^+^ and IFNγ^+^/IL-2^+^. Importantly, a similar tendency was observed when only considering paired longitudinal samples before and after second exposure (**Figures S7**, **S8** for spike- and CEFX- specific T-cell response, respectively).

**Figure 4:**
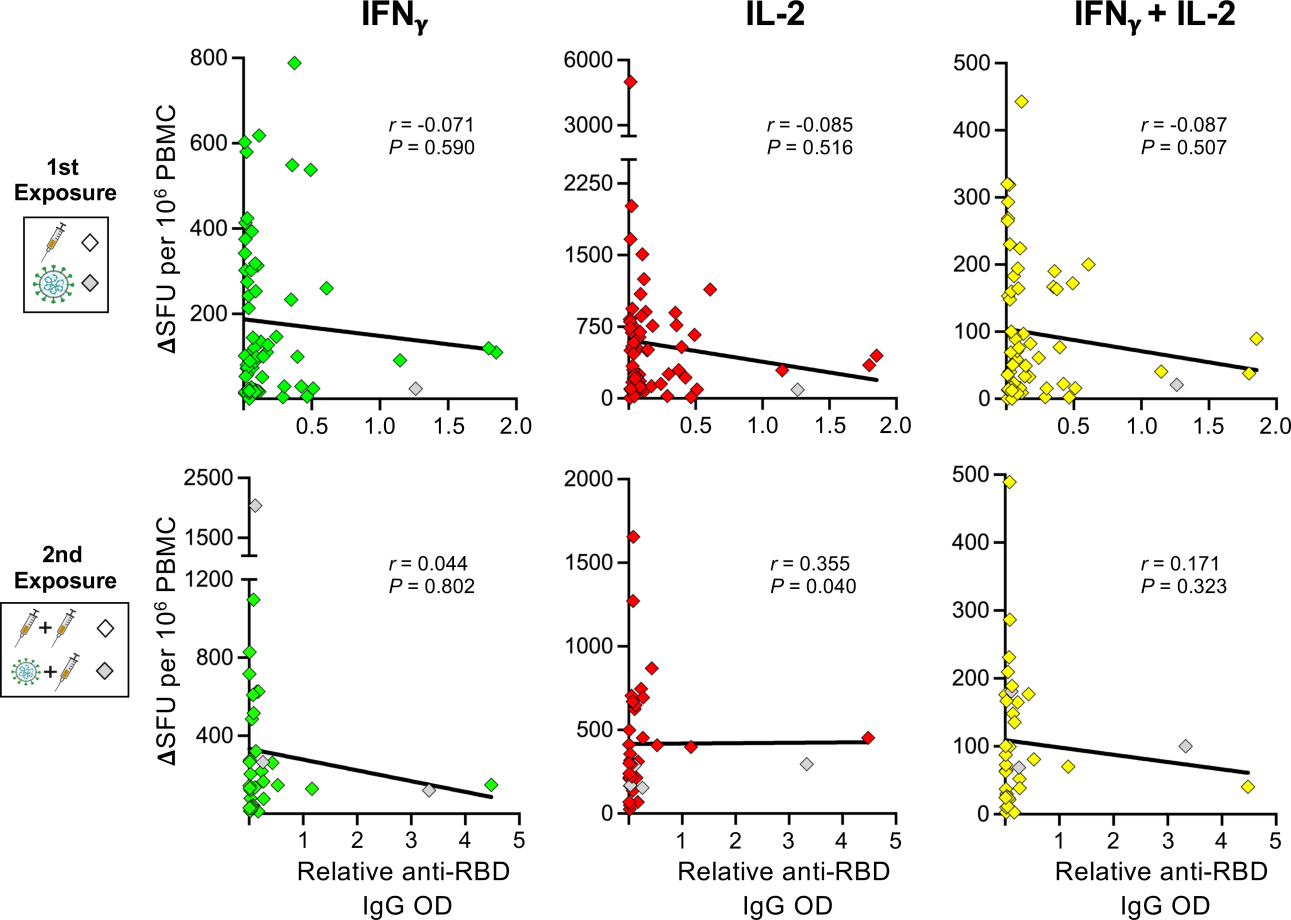
Correlation analysis between spike-specific cytokine^+^ T-cell and humoral responses in aCD20-MS patients. Spearman’s correlation between IFNγ^+^(green)-, IL-2^+^(red)-, and IFNγ^+^/IL-2^+^(yellow)-secreting T-cells (expressed as ΔSFU per 10^6^ PBMC) and the relative anti-RBD IgG OD values of aCD20-MS patients after first (n = 61) and second (n = 35) exposure. Correlation coefficient (r) and P value are included for each data set. The continuous bold black lines indicate regression lines.

Lastly, several studies have found an inverse correlation between humoral and T-cell responses in B-cell depleted aCD20-MS patients receiving mRNA-based COVID-19 vaccines (53, 65, 66), suggesting that compensatory mechanisms might be taking place. To determine if a similar association occurs in the current study, correlation analyses between the relative RBD IgG OD values and number of cytokine^+^-secreting T-cells from aCD20-MS patients before and after antigen re-exposure were performed. As presented in **Figure 4**, although our aCD20-MS cohort showed a clear discordant adaptive immune response after first and second exposures with several antibody-negative patients presenting strong T-cell response, no significant negative association was found between the humoral and cellular arms.

### 3.5 Conserved T-cell response against spike protein of SARS-CoV-2 omicron VOC

The capacity of the highly mutated SARS-CoV-2 omicron VOC to subvert the humoral response induced by vaccination with the ancestral spike protein has been well documented (60, 67–69). In contrast, the mutations accumulated in this variant confer only limited evasion from vaccine-induced T-cell reactivity due to the conservation of CD4^+^ and CD8^+^ T-cell epitopes (49–51, 60, 70–72). Since a strong T-cell response after primary vaccination series and sustained levels after re-exposure were found against the ancestral-spike protein in aCD20-MS patients, we decided to also look at conservation of cellular responses against the omicron version.

**Figure 5:**
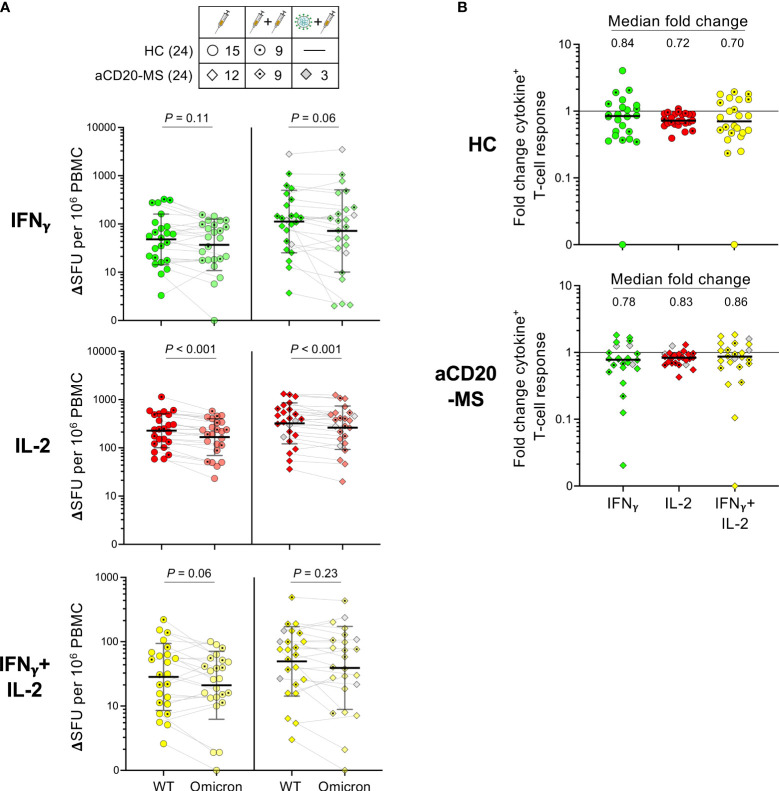
Ancestral compared to omicron spike-specific IFNγ^+^, IL-2^+^, and IFNγ^+^/IL-2^+^ T-cell responses in HC and aCD20-MS cohorts. **(A, B)** PBMCs were stimulated in parallel with 15-mer CoV-2 ancestral (Wuhan-Hu-1)- and omicron (BA.2)- derived spike peptide pools for 24 hours at 37 °C. A FluoroSpot assay was used to determine the number of spike-specific IFNγ^+^(green)-, IL-2^+^(red)-, and IFNγ^+^/IL-2^+^(yellow)-secreting T-cells (expressed as ΔSFU per 10^6^ PBMC). See also **Figure S9**. **(A)** Pairwise analysis of cytokine+ T-cell responses for HC (left, n = 24) and aCD20-MS patients (right, n = 24) after PBMC stimulation with ancestral (WT)- and omicron-spike peptide pools. Number of individuals included per type of exposure are indicated in top table. Data are presented as geometric mean (black line) ± geometric S.D., and significant P values between stimulations were calculated using a two-tailed Wilcoxon paired test. **(B)** Fold change in the frequencies of spike-specific cytokine^+^ T-cells between ancestral and omicron responses for HC (top) and aCD20-MS patients (bottom). Black horizontal line in each data set indicates median values.

A FluoroSpot assay was used to determine the T-cell response against omicron spike protein induced by antigen exposure in the study cohorts considering samples after first and second exposures (see **Figure S9** for representative FluoroSpot data). Samples from individuals infected during the beginning of omicron predominance at the end of 2021 were excluded from this analysis. As observed in **Figure 5A**, a reduction in the number of monofunctional IFNγ^+^-, IL-2^+^-, and polyfunctional IFNγ^+^/IL-2^+^-secreting T-cells was observed in HC and aCD20-MS after omicron (BA.2) spike-specific *ex vivo* stimulation compared to the corresponding ancestral-specific response, reaching only significant difference in the case of IL-2 (P< 0.001). Median relative frequencies of 84% (IFNγ^+^), 72% (IL-2^+^), and 70% (IFNγ^+^/IL-2^+^) for HC, and 78%, 83%, and 86% for aCD20-MS, were found for spike-specific cytokine^+^-secreting T-cells cross-recognizing BA.2 (**Figure 5B**). In conclusion, the T-cell response against omicron VOC is relatively conserved in both HC and aCD20-MS cohorts exposed to ancestral CoV-2 spike, suggesting similar T-cell response breadth.

## 4 Discussion

Multiple sclerosis patients treated with certain immunomodulatory medications can develop a severely compromised immune response to microbial infections and vaccines. Indeed, anti-CD20-treated MS patients with highly diminished B-cell counts and potential for failed anti-spike specific seroconversion are known to be more susceptible to severe disease and death induced by SARS-CoV-2 infection. For this group, the level of immune protection provided by current vaccines against symptomatic infections remains largely unknown, although a higher rate of breakthrough infections and longer viral incubation periods have been detected (30–33). This is to be expected since an absence of neutralizing antibodies impedes the effective blockage of virions during the initial stages of infection. Even though antibodies are also needed at later times during progression of infection, the role for T-cell responses is essential to restricting virus amplification which can lead to further tissue damage and severe disease.

As previously observed (25, 36, 53), we found a severely compromised spike-specific seroconversion in the aCD20-MS cohort compared to a healthy control group after a single CoV-2 antigen exposure (either through natural infection or after primary COVID-19 vaccination series). Only a maximum of 17.5% aCD20-MS positive responders containing anti-RBD antibodies with RBD-hACE2 blocking activity were detected after first exposure. Follow-up analysis of samples post-first exposure also highlights heterogenous humoral decay kinetics among positive responders. Interestingly, we observed a delayed humoral priming for some patients with a positive signal only in the later longitudinal time points (also observed for the second exposure cohort), although the possibility of an unreported asymptomatic infection occurring between dates of sample collection cannot be discarded. In addition, our longitudinal approach allowed for the detection of a limited priming induction effect after multiple exposures in the aCD20-MS group. In fact, even though an increase in the number of responders was observed after second exposure (up to 29.3%), the anti-RBD IgG antibody titers were much lower compared to recall levels observed in healthy controls (-31.5X geometric mean fold lower). Similar limited humoral responses have been found after the application of vaccination boosters in other aCD20-MS cohorts (73, 74), and in patients with hematological cancers treated with BCDTs (75, 76). Importantly, no correlation between antibody responses and the time span between vaccination and last anti-CD20 infusion was found in aCD20-MS patients after first exposure, suggesting limited spike-specific humoral priming within the treatment schedules considered in this study. Considering that only a small percentage of aCD20-MS patients received their first (10.4%) and second (22%) exposures past the recommended 6 month infusion regimen, and only one (0.7%; 1st exposure) and three (2.8%; 2nd) after 8 months, the low rates of seroconversion found in this study might be expected. This is also reflected in the low number of patients with CD19^+^ absolute count levels above 20 cells/μl or considered within normal range as measured in blood sample analysis done before infusion dates (7.1% for first and 3.7% for second exposure groups).

Besides diminished seroconversion after initial exposure, 11 out of 12 positive aCD20-MS responders after first exposure and three out of four aCD20-naïve MS patients who began treatment after this exposure were not capable of developing an anamnestic humoral immune response following antigen re-challenge. Overall, these results highlight a sustained failure in the initiation of an antibody response after COVID-19 exposure, as well as limited recall responses induced by re-exposure, in most patients on active anti-CD20 therapy. To facilitate seroconversion, a longer time span is needed between anti-CD20 infusion and vaccination which would allow for an increased incidence of B-cell repopulation (40, 77). To that end, in addition to monitoring B-cell count numbers, two studies have also shown the possibility of measuring therapeutic antibody concentrations in blood as a valuable baseline reference for determining the likelihood of seroconversion after COVID-19 vaccination in aCD20-MS patients (78, 79).

In contrast to the humoral response, aCD20-treated MS patients with B-cell depletion present a strong T-cell response which is shown on average to be higher than that of healthy controls. We find this to be true after first exposure and it remained higher after re-exposure (3.5X to 3.9X before, and 1.4X to 3.2X after re-challenge, depending on the cytokine^+^-releasing T-cells under consideration). Moreover, cellular responses against VOC omicron are largely conserved in aCD20-MS patients at levels comparable to HC individuals. For both cohorts, we found an anti-omicron T-cell response preservation of around 70 to 86% of the levels observed against the ancestral spike protein, similar to published data obtained from immunocompetent individuals (49, 71). Even though only the spike protein from sublineage BA.2 of omicron was used in this analysis, recent *in silico* data highlight preserved epitope recognition for more recent omicron sublineages including XBB and BQ.1 (80), suggesting that the level of conservation of T-cell responses in individuals vaccinated with ancestral spike is probably also maintained against these viruses.

The data presented here, together with our previous observation of a high-affinity response and expandability of spike-specific T-cells from aCD20-MS patients receiving their first COVID-19 vaccine primary series (57), represent evidence of a strong T-cell activation after exposure and suggest a partial level of protection even in severely B-cell depleted patients that failed to seroconvert. Previous publications have observed a similar discordant adaptive immune configuration occurring in aCD20-MS patients (38, 53–56, 65). Curiously, evidence of a heightened cellular response to COVID-19 vaccination has also been detected in other congenital or acquired conditions characterized by strong B-cell aplasia, including patients with B-cell malignancies treated with CD19-targeting chimeric antigen receptor (CAR) T-cell therapy (81, 82), and individuals with X-linked agammaglobulinemia (XLA) who have an inherited inability to generate mature B-cells (83–85). Overall, these examples indicate a complex immunological response to SARS-CoV-2 infection and COVID-19 vaccination and suggest the potential of compensatory adaptive immunological outputs in the context of strong B-cell deficiency. Further characterization is needed to better understand the contribution of T-cells in vaccine immune protection. For example, a higher accumulation of nasal resident T-cells has been noticed in vaccinated healthy individuals after a breakthrough infection (86). As evidence of an efficient localization of functional T-cells in relevant tissues in aCD20-MS patients, a similar study could be of interest for these individuals.

Interestingly, a few publications have detected an inverse correlation between both adaptive immune arms for COVID-19 vaccinated aCD20-MS patients (53, 65, 66), suggesting compensatory immune processes might be taking place. In fact, a tendency to a higher T-cell response in patients with lower anti-RBD IgGs levels was observed in our correlation studies, although not statistically significant. Mechanisms that could potentially explain this phenomenon, maybe occurring in an MS disease-specific manner, include i) higher presence of pre-existing cross-reactive memory T-cells with potential to contribute to COVID-19 vaccine-induced immune responses, ii) increased occurrence of a specific HLA configuration, iii) indirect rearrangement of T-cell compartments during treatment that favors a stronger T-cell response after *de novo* antigen exposure, iv) elimination of a CD20-containing cell with an inhibitory role against T-cell response, and v) lack of antibody-mediated neutralization of vaccine-induced spike protein contributing to a more efficient priming of antigen-specific T-cells.

The results from this study have implications in health care strategies and vaccine design and application. In terms of types of vaccines that could potentially be applied in the context of severe B-cell depletion, our work suggests aCD20-MS patients with a high probability of not seroconverting might benefit from a vaccine configuration designed for an improved T-cell response. An example of this concept is the peptide-based CoVac-1 vaccine recently described and currently being tested in clinical trials for protection of B-cell deficient individuals (87) (ClinicalTrials.gov Identifier: NCT04954469). An advantage of using peptide vaccines for T-cell specific stimulation is the possibility of incorporating other CoV-2 proteins besides spike protein, thus allowing for a broader response.

Lastly, to understand how COVID-19 immunizations protect aCD20-MS patients who failed to seroconvert but maintained an efficient T-cell response, we need to better characterize their real-world, vaccine-induced immune protection against SARS-CoV-2. More breakthrough infections have been observed in aCD20-MS patients due to the lack of antibody-mediated responses. Whether this is followed by a greater incidence of severe disease or death, or, on the other hand, enough protection is provided by the functionally strong cellular response described in this and other studies (or by other immune responses that remain unaltered) needs to be closely monitored. Furthermore, prophylactic use (or prompt use in the event of infection) of VOC-efficient therapeutic anti-spike neutralizing antibodies, easy access to COVID-19 testing and oral antivirals, and the implementation of extra precautions remain important therapeutic approaches for preventing CoV-2 infections and complications in B-cell depleted MS patients.

## 5 Limitations

There are several limitations associated with this study. First, this is a single-center study, so it is subject to potential bias. Additional analysis should be carried out to contextualize the conclusions drawn here. Second, even though the humoral response analysis in this study benefited from the use of two different serological assays, the results obtained using a surrogate assay for the detection of anti-RBD IgG-mediated neutralizing activity might not faithfully reflect the results from live virus neutralization assays, which are considered the gold standard. Third, the use of frozen PBMCs in T-cell experiments can have an impact on the assay’s results (88, 89). In addition, the FluoroSpot assay used for the detection of spike-specific cellular responses is less sensitive than others (e.g., flow cytometry-based detection assays like Intracellular Cytokine Staining (ICS) and Activation-Induced Markers (AIM)) and does not allow for the differentiation among T-cell subpopulations. Fourth, even though a categorization based on immunological experiences was attempted in this study, it should be stated that different immune responses with different functionalities and anatomical locations are at play when considering exposures based on vaccination, infection, or a combination of both. Further studies are needed to better characterize the immune responses in individuals within specific exposure groups. Fifth, the study mostly includes patients receiving COVID-19 exposure less than 6 months after their last anti-CD20 infusion. Thus, a higher prevalence of B-cell depleted patients is apparent which limited our capacity to analyze events of seroconversion in the context of delayed anti-CD20 treatment and B-cell repopulation. Lastly, the healthy control group used in this study was of a significantly younger age than the aCD20-MS cohort and received as first exposure almost exclusively primary vaccination series with BNT162b2/Cominarty.

## Data availability statement

The raw data supporting the conclusions of this article will be made available by the authors, without undue reservation.

## Ethics statement

The studies involving human participants were reviewed and approved by WIRB (Western Institutional Review Board). The patients/participants provided their written informed consent to participate in this study.

## Author contributions

Conceptualization, RA-D, JLin, and SAS. Methodology, RA-D, JLin, JLei, JLiu, MR, AO, AR, AK, VK, GF, MM, and SAS. Investigation, RA-D, JLin, and SAS. Writing – Original Draft, RA-D. Writing – Review & Editing, RA-D, JLin, JLei, JLiu, MR, AO, AR, AK, VK, GF, MM, and SAS. Funding Acquisition, SAS. Supervision, RA-D, JLin and SAS. All authors contributed to the article and have seen and approved the submitted version.
