# Supplementary material for: Humoral and cellular responses to repeated COVID-19 exposure in multiple sclerosis patients receiving B-cell depleting therapies: a single-center, one-year, prospective study
Source: Front Immunol. 2023 Jun 28;14:1194671. doi: 10.3389/fimmu.2023.1194671 (PMC10338057; doi:10.3389/fimmu.2023.1194671)
Supplement: Supplementary file 1 [file Image_1.pdf]

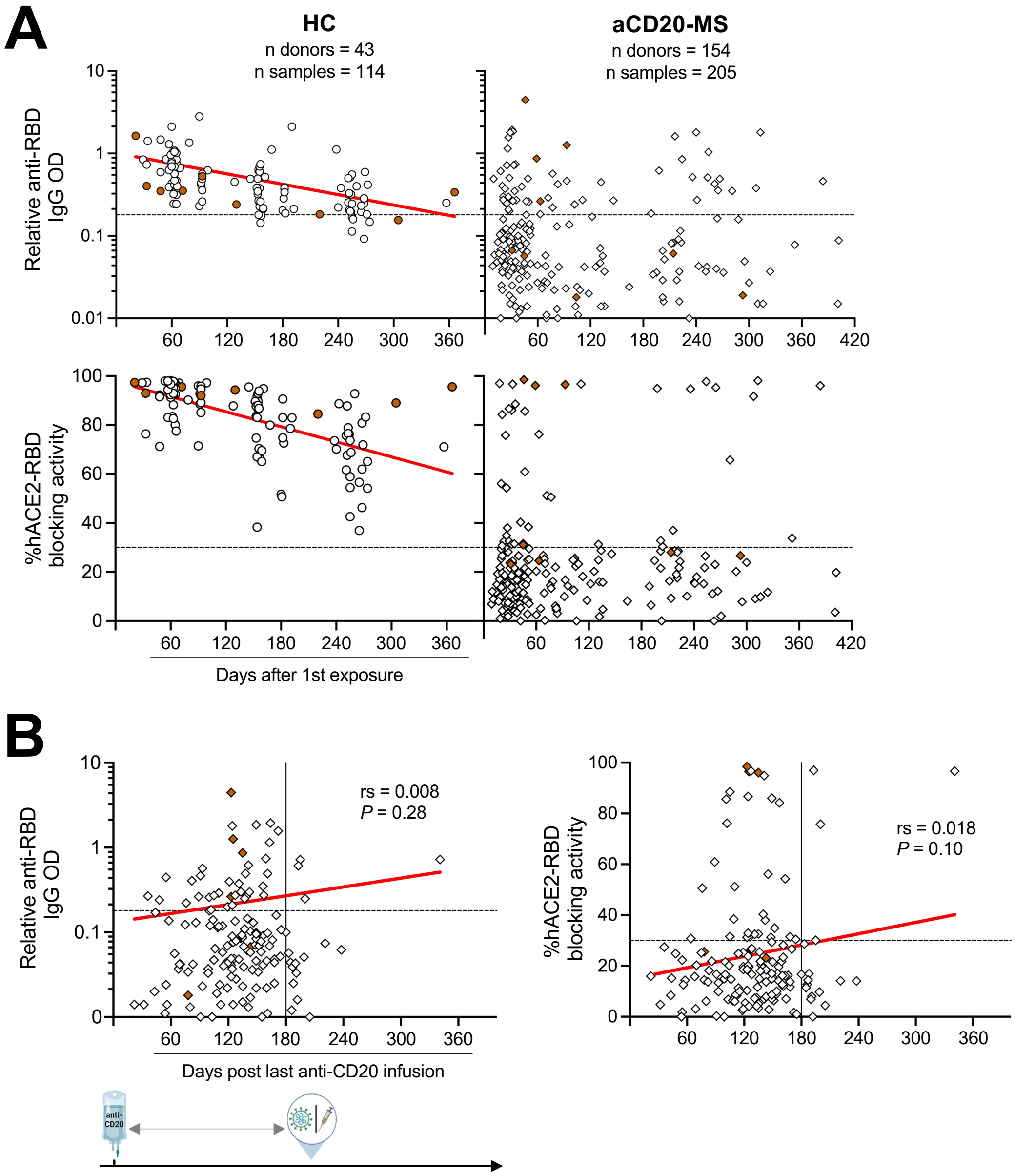

Figure S1. Additional characterization of the humoral response to 1st COVID-19 exposure: decay kinetics and time since last anti-CD20 infusion.

A) Cross-sectional presentation of relative anti-RBD IgG OD values (top) and percentage hACE2-RBD blocking activity (bottom) in HC (114 samples from 43 donors) and aCD20-MS individuals (205 samples from 154 donors). For HC, best non-linear fit lines (red) showing decay kinetics are presented. Pearson correlation analysis (relative anti-RBD IgG OD: R = -0.427, P< 0.001; %hACE2-RBD blocking activity: R = -0.602, P< 0.001). Color code of symbols is same as in Figure 1.

B) Lack of significant correlation between humoral response and last anti-CD20 infusion to 1st exposure interval in aCD20-MS patients. Relative anti-RBD IgG OD values (left) and percentage hACE2-RBD blocking activity (right) plotted against time span between last anti-CD20 infusion and first antigen exposure dates. Only first samples collected after exposure were considered in the analysis. Rs coefficient and P values from simple linear regression analysis are indicated inside graphs. A solid vertical line at 180 days (current recommended time interval between anti-CD20 infusions for MS disease treatment) is shown as reference.

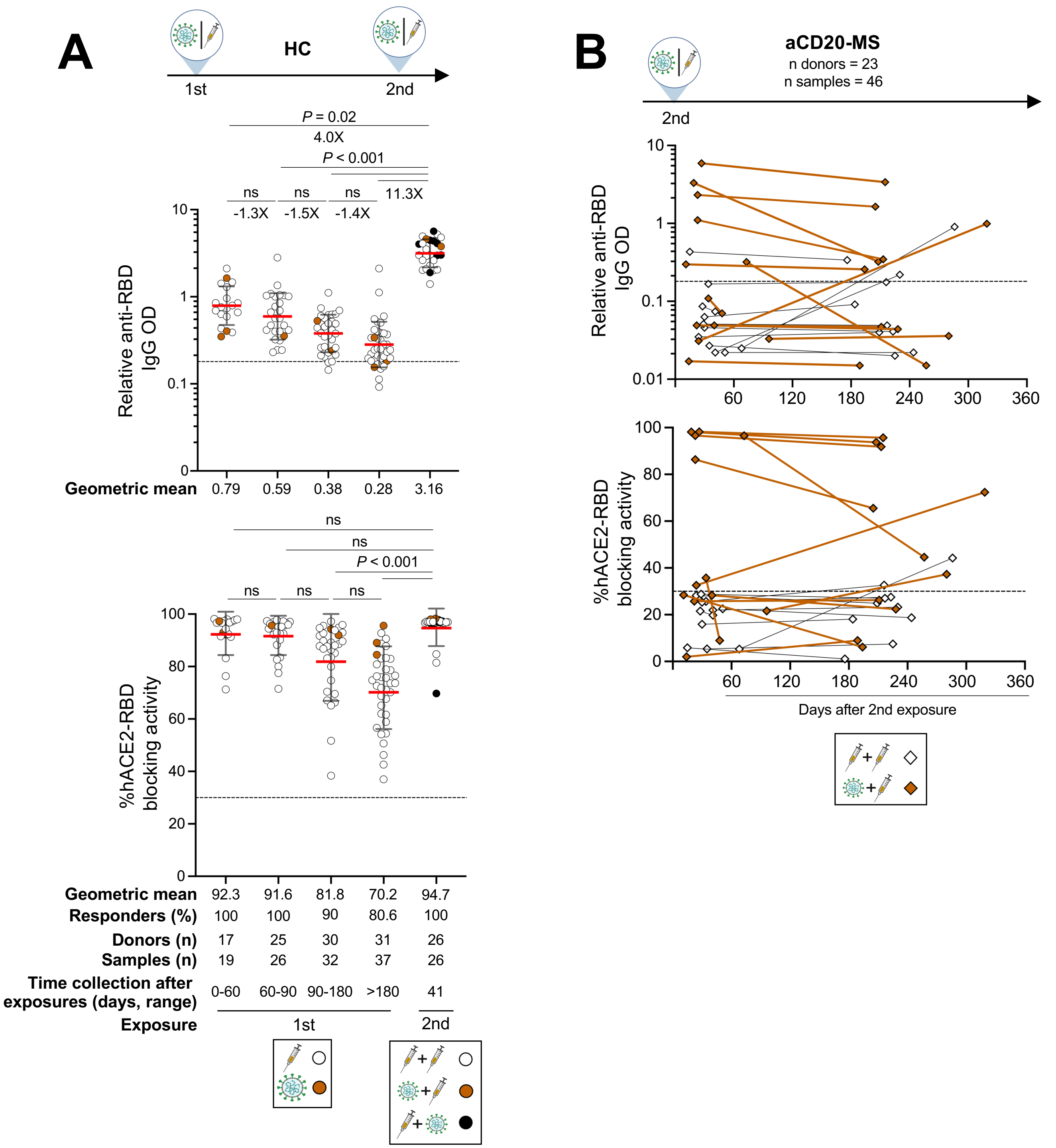

Figure S2. Additional characterization of the humoral response to 2nd COVID-19 exposure.

A) Decay kinetics of the humoral response after 1st COVID-19 exposure and immune recall effect after 2nd exposure in HC individuals. Anti-RBD IgG OD values (top) and percentage hACE2-RBD blocking activity (bottom) were grouped based on sample collection times after exposures (0-60, 60-90, 90-180 and > 180 days after 1st exposure; 41 median days after 2nd exposure). Data is presented as geometric mean (red line) with geometric S.D., and differences between groups were analyzed using the Kruskal-Wallis test. Number of donors and samples, and percentage of individuals with positive humoral response are indicated below the graphs. Significant P values and fold changes are shown above each data set. Color code of symbols is same as in Figure 2.

B) Decay kinetics of the humoral response after 2nd COVID-19 exposure in aCD20-MS (46 samples from 23 donors). Longitudinal presentation of relative anti-RBD IgG OD values (top) and percentage hACE2-RBD blocking activity (bottom). Each figure dot represents a single sample with 2 antibody measurements per donor after 2nd exposure. Longitudinal paired samples are linked with a solid line.

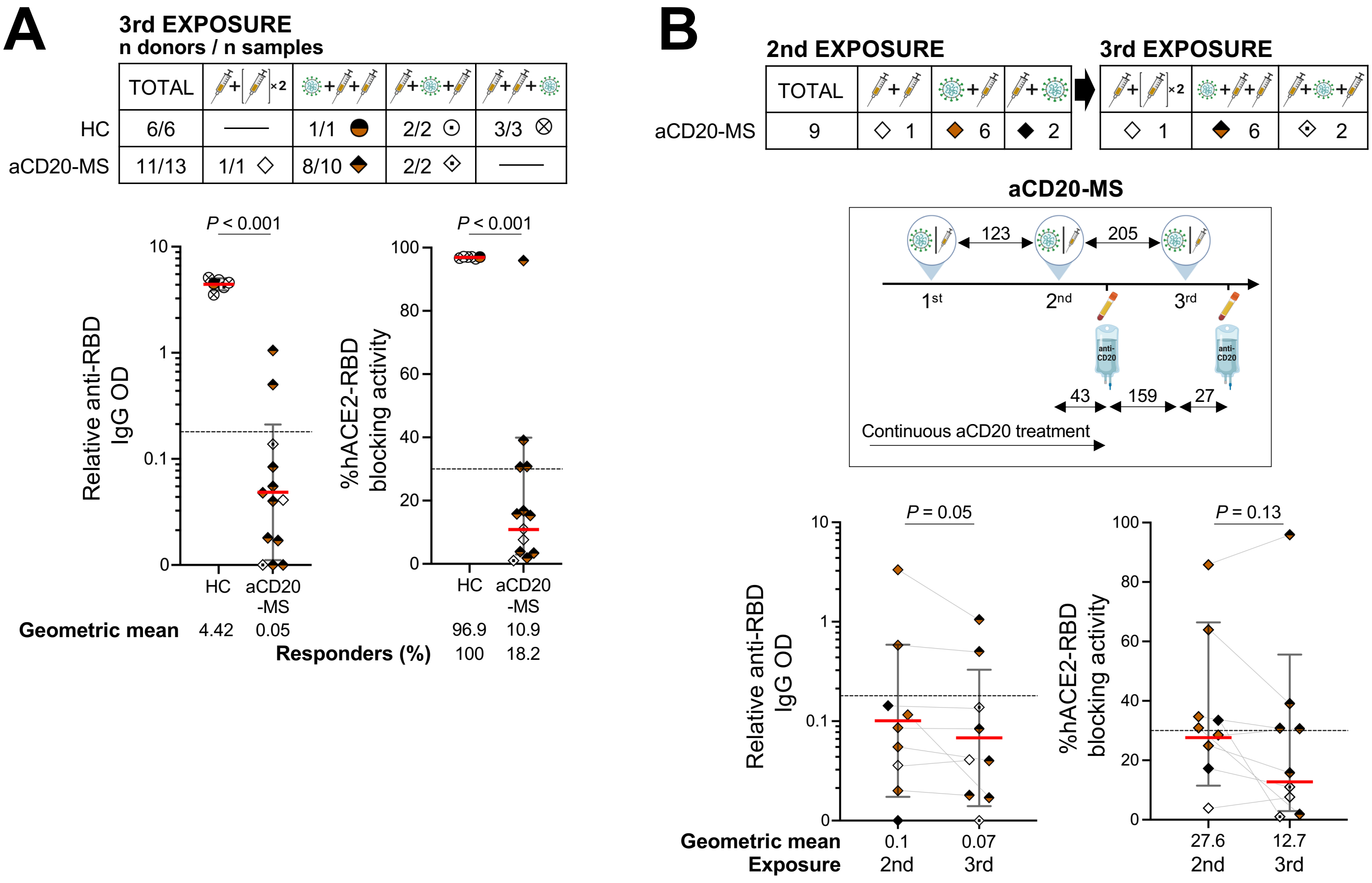

Figure S3. Deficient humoral response to 3rd CoV-2-spike protein exposure in aCD20-MS patients.

A) Distribution of total donors and samples based on COVID-19 exposure profile (top table), and relative RBD IgG OD values (left graph) and percentage hACE2-RBD blocking activity (right graph) after a third antigen exposure in HC (n donors = 6/n samples = 6) and aCD20-MS patients (n donors = 11/n samples = 13) (see also Table S1). Data from grouped samples is presented as geometric mean (red line) with geometric S.D., and differences between cohorts were calculated using a Mann-Whitney U test. Percentages of individuals with positive humoral response are indicated below right graph and significant P values are shown above each data set.

B) Analysis of the humoral response before and after 3rd COVID-19 exposure in aCD20-MS patients using paired longitudinal samples (n donors = 9). Top tables include total donors organized based on COVID-19 exposure profile. Illustration depicts the timescale of exposures, sample collections and anti-CD20 infusions (numbers indicate median days for each time lapse). Bottom graphs show relative RBD IgG OD values (left) and % RBD-hACE2 blocking activity (right) before and after a third exposure to CoV-2 spike protein. Data is presented as geometric mean (red line) with geometric S.D., and differences between 2nd and 3rd exposure were calculated using a two-tailed Wilcoxon paired test.

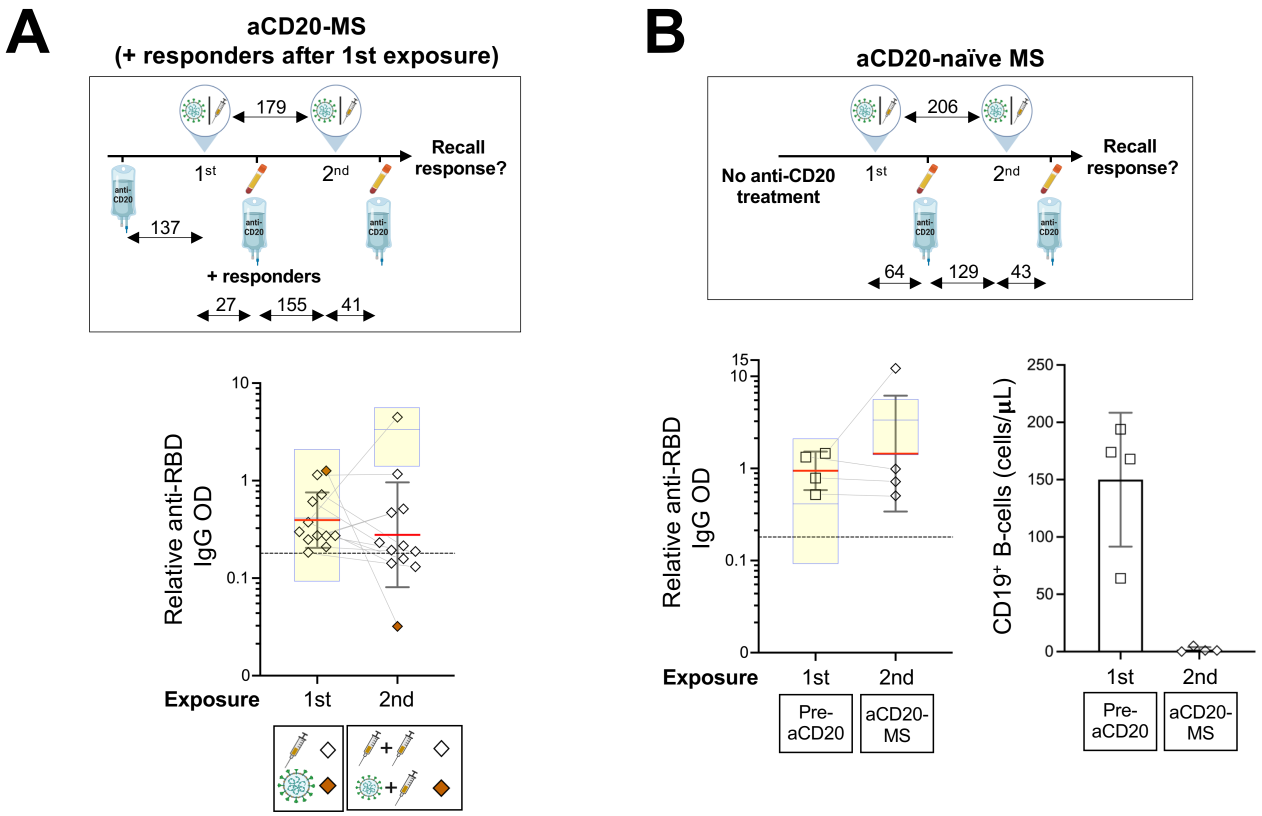

Figure S4. Deficient humoral recall response to CoV-2 spike protein re-exposure in seroconverted aCD20-MS and treatment-naïve patients.

A, B) Serum samples from MS patients with continuous anti-CD20 treatment and positive humoral response after 1st exposure (aCD20-MS; n donors = 12) or receiving 1st infusion after vaccination (aCD20-naïve MS; n donors = 4), were followed up for assessment of antibody-based humoral recall response after antigen re-exposure. Diagrams illustrate the timescale of exposures, sample collections and anti-CD20 infusions (numbers indicate median days for each time lapse).

A) Relative anti-RBD IgG OD values before and after 2nd CoV-2 spike antigen exposure in aCD20-MS patients with positive humoral response after 1st exposure. Geometric mean (red lines) with geometric S.D. is indicated for each data set. As reference, yellow boxes with blue line borders inside graphs indicate minimum-to-maximum and mean values observed in healthy controls before and after 2nd exposure (see Figure 2).

B) Relative anti-RBD IgG OD values before (squares) and after (diamonds) 2nd antigen exposure in aCD20-naïve MS patients. B-cell count numbers (cells/µL) as measured in blood analysis performed close to infusion/collection dates are shown in the right graph with box plots indicating mean with S.D.

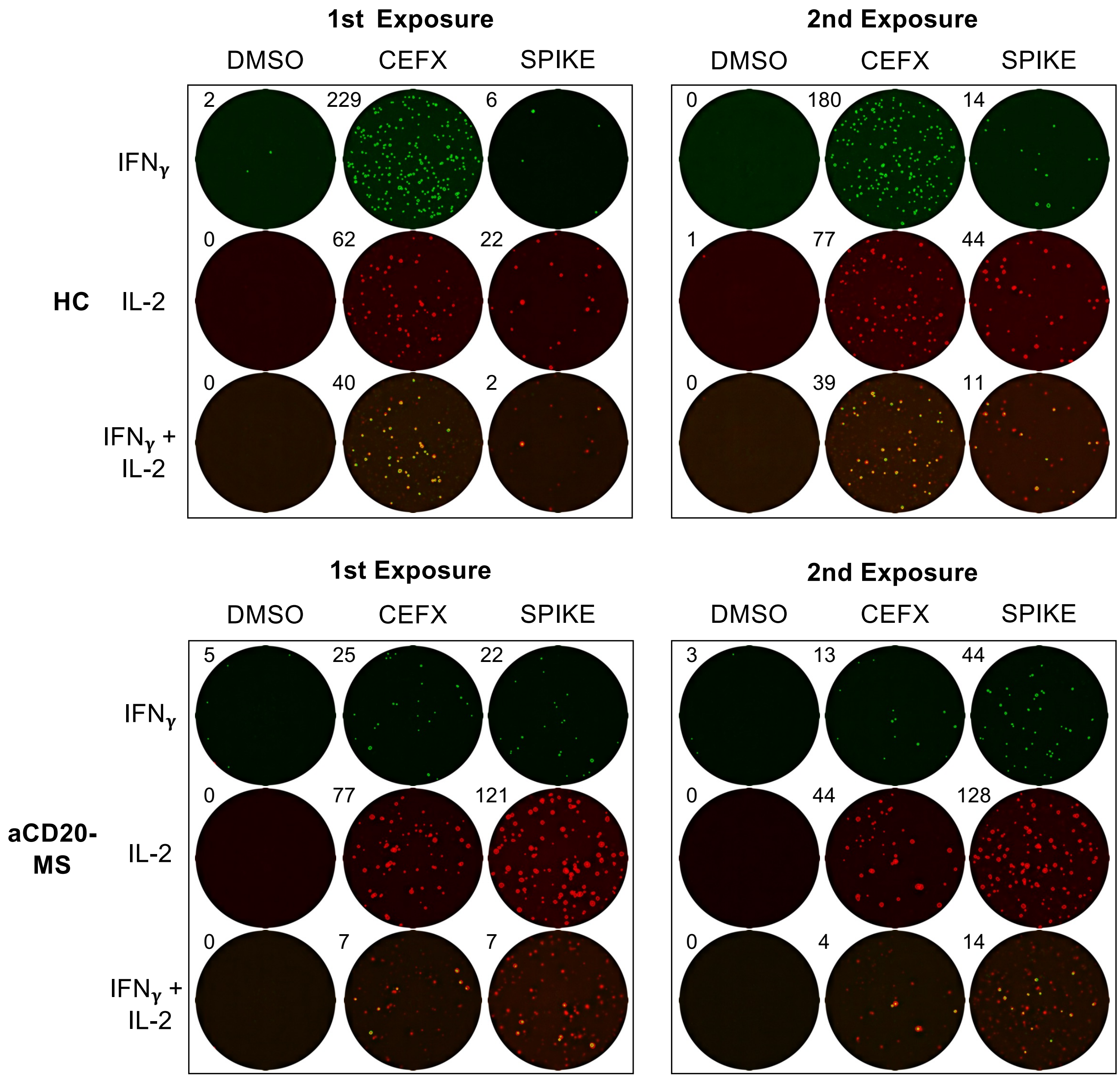

Figure S5. Representative IFNγ^{+}, IL-2^{+} and IFNγ^{+}/IL-2^{+} FluoroSpot data after stimulation of PBMCs isolated from one healthy control individual and one aCD20-MS patient before and after 2nd COVID-19 exposure with an ancestral-based spike peptide pool.

Related to **Figure 3**. FluoroSpot wells scanned with an ImmunoSpot® reader showing ancestral spike-specific IFNγ^{+}(green)-, IL-2^{+}(red)- and IFNγ^{+}/IL-2^{+}(yellow)-positive T-cells after a 24-hour *ex vivo* peptide pool incubation. For each selected PBMC sample, a negative (0.4% DMSO) and positive (CEFX peptide pool) control stimulations were also included.

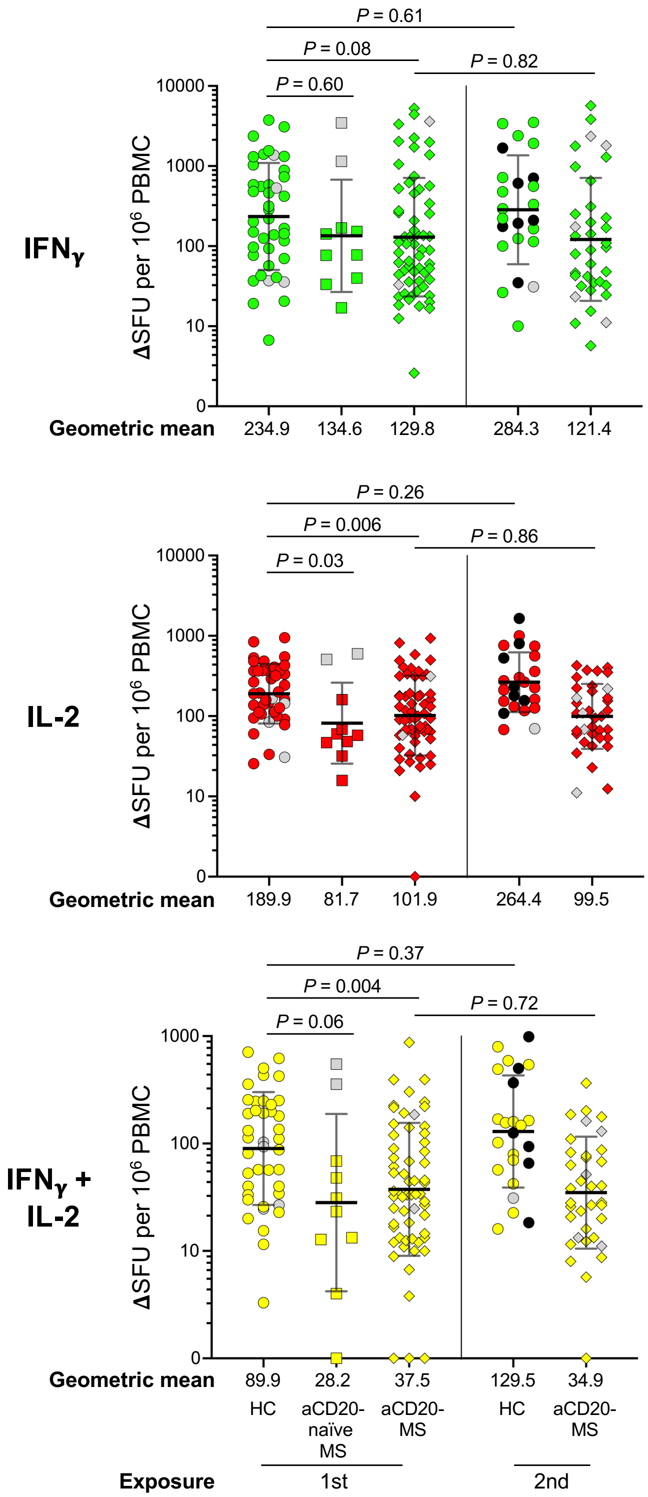

Figure S6. CEFX-specific IFNγ⁺, IL-2⁺ and IFNγ⁺/IL-2⁺ T-cell responses after 1st and 2nd COVID-19 exposure in aCD20-MS cohort compared to HC.

Related to **Figure 3**. FluoroSpot quantification of CEFX-specific $IFN\gamma^+$(green)-, $IL\text{-}2^+$(red)- and $IFN\gamma^+/IL\text{-}2^+$(yellow)-secreting T-cells using PBMC samples from HC and aCD20-MS patients after 1st and 2nd COVID-19 exposure, and aCD20-naïve MS after 1st exposure. Data is presented as geometric mean of ΔSFU per 10^6 PBMC (black line) with geometric S.D., and differences between cohorts after 1st exposure were analyzed using the Kruskal-Wallis test. Differences between cohorts after 2nd exposure and between exposures within cohorts were analyzed using the Mann-Whitney U test.

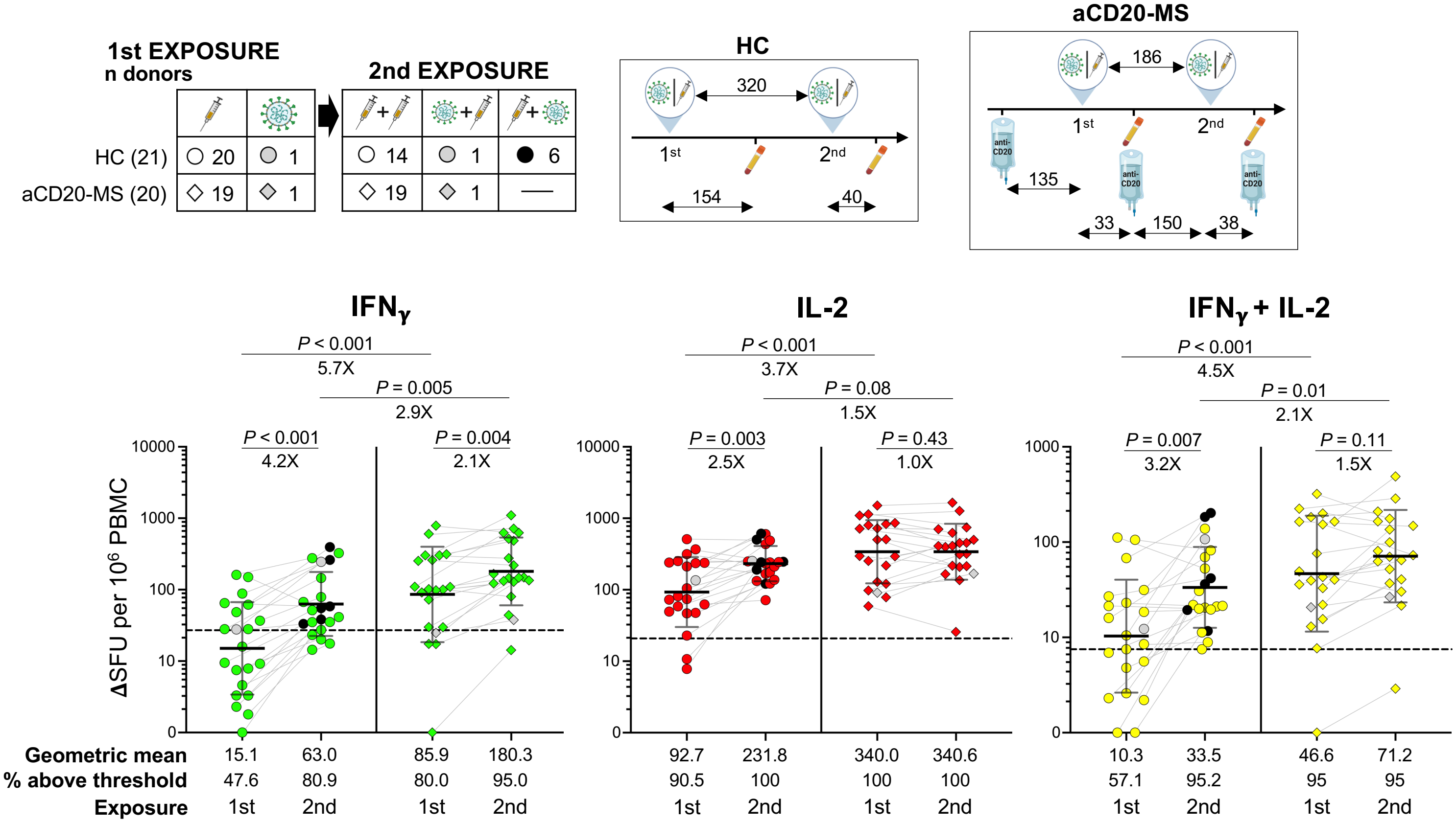

Figure S7. Ancestral spike-specific IFNγ^{+}, IL-2^{+} and IFNγ^{+}/IL-2^{+} T-cell responses before and after 2nd COVID-19 exposure in aCD20-MS cohort compared to healthy controls using paired longitudinal samples.

Longitudinal analysis of spike-specific cytokine^{+}-secreting T-cells for HC (n donors = 21) and aCD20-MS patients (n = 20) before and after 2nd antigen exposure. Number of individuals included per exposure profile is indicated in top left table. Illustrations at top right depict timescale of exposures and sample collections for each group (numbers indicate median days for each time lapse). The fraction of individuals above threshold and the geometric means are indicated below the graphs, and P values and fold changes are shown above each data set. Data is presented as geometric mean (black line) ± geometric S.D., and differences between before and after re-exposure within cohorts were calculated using a two-tailed Wilcoxon paired test. Differences between cohorts were analyzed using the Mann-Whitney U test. See also Figure S8.

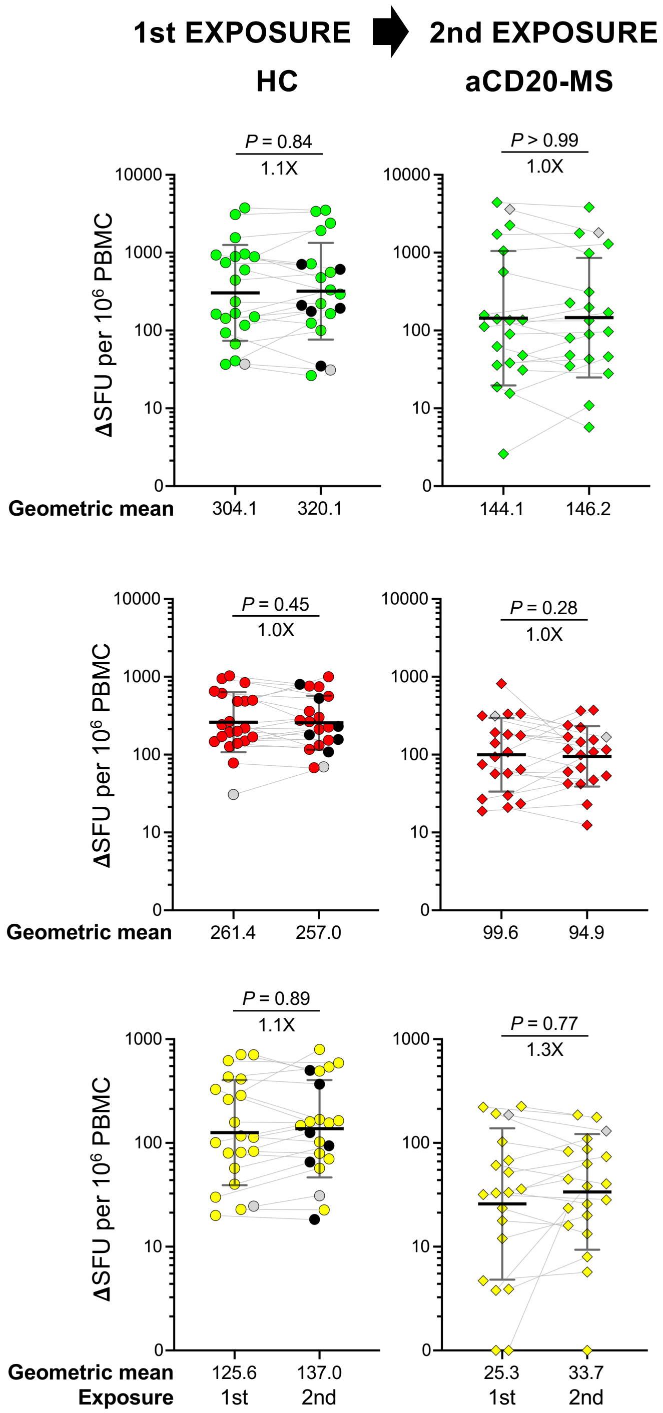

Figure S8. CEFX-specific IFNγ$^+$, IL-2$^+$ and IFNγ$^+$/IL-2$^+$ T-cell responses before and after 2nd COVID-19 exposure in aCD20-MS cohort compared to healthy controls using paired longitudinal samples.

Related to **Figure S7**. FluoroSpot quantification of CEFX-specific IFNγ$^+$(green)-, IL-2$^+$(red)- and IFNγ$^+$/IL-2$^+$(yellow)-secreting T-cells using paired longitudinal samples from HC (left) and aCD20-MS patients (right) before and after 2nd COVID-19 exposure. Data is presented as geometric mean of ΔSFU per 10^6 PBMC with geometric S.D., and differences between before and after re-exposure were calculated using a two-tailed Wilcoxon paired test. Significant P values and fold changes are indicated above each data set.

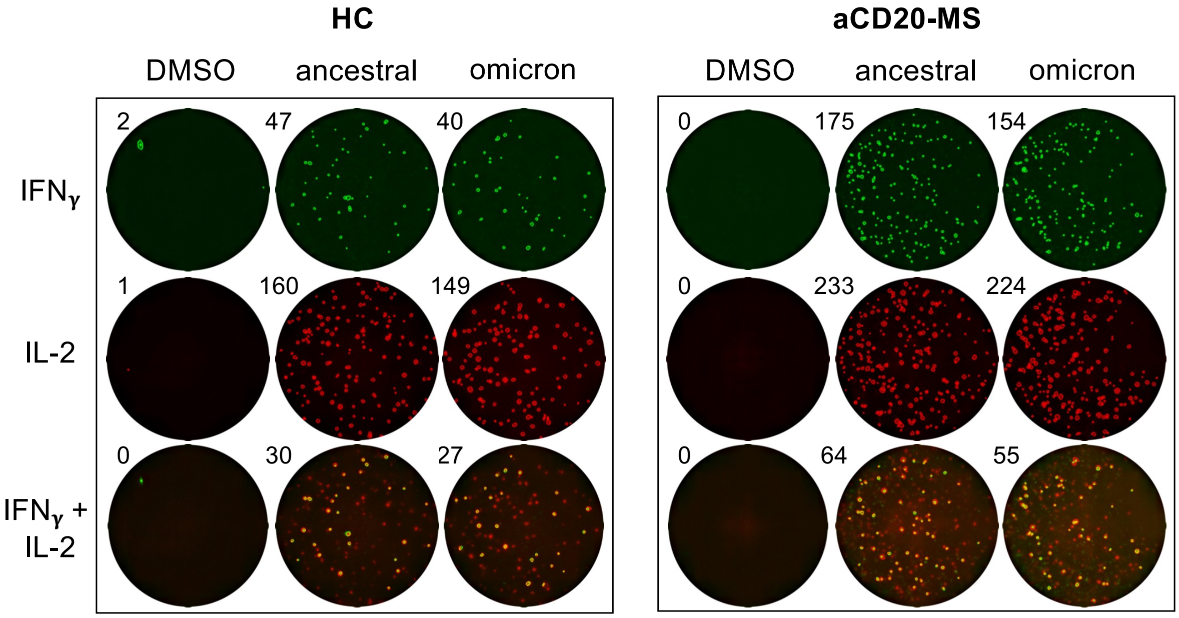

Figure S9. Representative IFNγ⁺, IL-2⁺ and IFNγ⁺/IL-2⁺ FluoroSpot data after stimulation of PBMCs isolated from one healthy control individual and one aCD20-MS patient with ancestral- or omicron-spike peptide pools.

Related to **Figure 5**. FluoroSpot wells scanned with an ImmunoSpot® reader showing ancestral- and omicron-spike specific IFNγ^{+}(green)-, IL-2^{+}(red)- and IFNγ^{+}/IL-2^{+}(yellow)-positive T-cells after a 24-hour *ex vivo* peptide pool incubation. For each selected PBMC sample, a negative (0.4% DMSO) control stimulation was also included.
