# Supplementary material for: Humoral and cellular responses to repeated COVID-19 exposure in multiple sclerosis patients receiving B-cell depleting therapies: a single-center, one-year, prospective study
Source: Front Immunol. 2023 Jun 28;14:1194671. doi: 10.3389/fimmu.2023.1194671 (PMC10338057; doi:10.3389/fimmu.2023.1194671)
Supplement: Supplementary file 2 [file Table_1.docx]

|  | **3rd EXPOSURE** | |
| --- | --- | --- |
|  | **HC** | **aCD20-MS** |
| **Donors, n** | 6 | 11 |
| **Samples, n** | 6 | 13 |
| **Age, mean years [range]** | 36.3 (23-59) | 55 (27-71) |
| **Gender, n (%)** |  |  |
| Male | 1 (16.7%) | 5 (45.4%) |
| Female | 5 (83.3%) | 6 (54.6%) |
| **MS type, n (%)** |  |  |
| RRMS |  | 7 (63.6%) |
| SPMS |  | 4 (36.4%) |
| PPMS |  |  |
| **anti-CD20 therapy, n (%)** |  |  |
| Ocrelizumab |  | 9 (81.8%) |
| Rituximab |  | 2 (18.2%) |
| **CD19+ absolute count > 20 cell/μl, n (%)** |  | 1 (9.1%) |
| **CD^+^ absolute count, mean cell/μl [reference range]** |  |  |
| CD3^+^ |  | 1835 [708-2662] |
| CD4^+^ |  | 1054 [410-1604] |
| CD8^+^ |  | 813 [137-1003] |
| **CD4^+^/CD8^+^ ratio [reference range]** |  | 2.8 [0.9-4.2] |
| **Vaccinated + 2 Boosters, n (%)** |  | 1 (9.1%) |
| **CoV-2 Infection and vaccination, n (%)** |  |  |
| Infected + Vaccinated + Booster | 1 (16.7%) | 8 (72.7%) |
| Vaccinated + Infected + Booster | 2 (33.3%) | 2 (18.2%) |
| Vaccinated + Booster + Infected | 3 (50%) |  |
| **Time intervals, median days [IQR]** |  |  |
| ^*^3rd exposure to collection | 28 [13-43] | 27 [22-44] |
| 1st exposure to last anti-CD20 infusion |  | 48 [28-144] |
| 2nd exposure to last anti-CD20 infusion |  | 142 [122-148] |
| 3rd exposure to last anti-CD20 infusion |  | 162 [129-168] |
| 1st to 2nd exposure | 271 [178-294] | 144 [102-332] |
| 2nd to 3rd exposure | 71 [33-184] | 196 [154-212] |
| **Longitudinal samples (n donors/ n samples)** |  |  |
| 3rd exposure without re-exposure |  | 2/4 |
| 2nd to 3rd exposure |  | 9/18 |

**Table S1. Clinical characteristics of study participants with third COVID-19 exposure.**

Clinical characteristics of healthy control individuals (HC) and MS patients on continuous anti-CD20 treatment (aCD20-MS) with 3rd exposure.

*For donors with more than one longitudinal point, only earliest point collected was considered.

IQR: interquartile range.
